# Advanced MRI Protocols to Discriminate Glioma From Treatment Effects: State of the Art and Future Directions

**DOI:** 10.3389/fradi.2022.809373

**Published:** 2022-04-15

**Authors:** Dania G. Malik, Tanya J. Rath, Javier C. Urcuyo Acevedo, Peter D. Canoll, Kristin R. Swanson, Jerrold L. Boxerman, C. Chad Quarles, Kathleen M. Schmainda, Terry C. Burns, Leland S. Hu

**Affiliations:** ^1^Department of Radiology, Mayo Clinic, Phoenix, AZ, United States; ^2^Mathematical Neurooncology Lab, Precision Neurotherapeutics Innovation Program, Mayo Clinic, Phoenix, AZ, United States; ^3^Departments of Pathology and Cell Biology, Columbia University, New York, NY, United States; ^4^Department of Diagnostic Imaging, Brown University, Providence, RI, United States; ^5^Department of Neuroimaging Research & Barrow Neuroimaging Innovation Center, Barrow Neurologic Institute, Phoenix, AZ, United States; ^6^Department of Biophysics & Radiology, Medical College of Wisconsin, Milwaukee, WI, United States; ^7^Departments of Neurologic Surgery and Neuroscience, Mayo Clinic, Rochester, MN, United States

**Keywords:** post-treatment, glioblastoma, MRI, response assessment, advanced, perfusion, metabolic

## Abstract

In the follow-up treatment of high-grade gliomas (HGGs), differentiating true tumor progression from treatment-related effects, such as pseudoprogression and radiation necrosis, presents an ongoing clinical challenge. Conventional MRI with and without intravenous contrast serves as the clinical benchmark for the posttreatment surveillance imaging of HGG. However, many advanced imaging techniques have shown promise in helping better delineate the findings in indeterminate scenarios, as posttreatment effects can often mimic true tumor progression on conventional imaging. These challenges are further confounded by the histologic admixture that can commonly occur between tumor growth and treatment-related effects within the posttreatment bed. This review discusses the current practices in the surveillance imaging of HGG and the role of advanced imaging techniques, including perfusion MRI and metabolic MRI.

## Introduction

Treatment of high-grade gliomas (HGGs) requires a multidisciplinary approach, with the standard of care first-line treatment comprising the combination of maximal safe surgical cytoreduction, followed by adjuvant radiotherapy and chemotherapy (1). Posttreatment surveillance imaging plays a vital role in assessing the therapeutic response and disease progression/recurrence to guide the clinical management and tailored therapy for each patient. Contrast-enhanced MRI (CE-MRI), with and without intravenous administration of a gadolinium-based contrast agent (GBCA), is considered to be the clinical benchmark for imaging follow-up (2). However, the use of CE-MRI alone can be limited in distinguishing the overlapping features of tumor progression and posttreatment-related effects. Specifically, both entities can present as contrast-enhancing lesions due to blood-brain-barrier (BBB) disruption with resultant vasogenic edema. Both entities can also progress over time and present as serially enlarging masses on follow-up imaging (3).

Multiple advanced imaging techniques including perfusion and metabolic MRI provide additional biomarkers of tissue characteristics that can improve the diagnostic specificity. Perfusion techniques include dynamic susceptibility contrast (DSC) MRI based on T2- or T2^*^-weighted susceptibility effects of intravascular contrast agent, to measure microvasculature features including microvessel volume. Dynamic contrast enhanced (DCE) MRI, another perfusion technique, can measure features such as vascular leakage based on T1-weighted (T1W) relaxivity effects related to BBB permeability, while arterial spin labeling (ASL) uses the magnetic labeling of arterial blood as an endogenous contrast agent to primarily measure blood flow. The use of perfusion MRI techniques relies on the premise that HGG recurrence will exhibit higher vascularity (microvessel volume, blood flow) compared to non-tumoral posttreatment effects. Magnetic resonance spectroscopy (MRS) is a form of metabolic imaging that can quantify specific metabolites (e.g., choline, lipid/lactate, and glutamine/glutamate) associated with tumor growth, necrosis, and posttreatment effects.

These techniques have been widely applied in the setting of posttreatment HGG, as evidenced by the myriad of published studies in this field of research. These techniques are also widely available in clinical practice, which speaks to their immediate clinical translation and impact. However, there are persisting obstacles to widespread adoption, including discrepancies and variability in the published thresholds/criteria for diagnosis and the perceived utility of each technique. These issues are multifactorial, but technical factors and the integrity of validation methods are likely contributors, particularly in the setting of intratumoral heterogeneity and histologic admixture. In this review, we critically assess the literature to date and discuss some of the contributing factors to this variability, including the differences in image acquisition, protocol design, and validation methods. We highlight the methodologies promoting consistency, accuracy, and robustness of advanced perfusion and metabolic imaging for HGG- response assessment. We also discuss the need for spatially resolved validation methods to overcome the challenges of intratumoral heterogeneity. Finally, we summarize the recent efforts in the development of consensus recommendations for the acquisition of perfusion MRI in HGG and discuss the needs moving forward for clinical implementation and widespread adoption.

## Current Treatment Practices

The current treatment of HGG consists of maximal safe surgical resection followed by adjuvant radiotherapy and chemotherapy. Radiotherapy has been used since the 1970s after survival benefit was seen with its use following surgical resection of HGGs (4). The Stupp protocol (5), published in 2005, showed that the addition of temozolomide (TMZ), a cytotoxic alkylating agent, to adjuvant radiotherapy further contributed to survival benefit. The Stupp protocol is currently the standard of care for glioblastoma (GBM) and consists of 6 weeks of concomitant adjuvant radiotherapy and chemotherapy with TMZ followed by 6 months of TMZ alone. Additional FDA-approved therapies include other cytotoxic alkylating agents (lomustine, intravenous carmustine, and carmustine wafer implants), bevacizumab, a vascular endothelial growth factor (VEGF) inhibitor, and an electric field therapy referred to as Tumor Treating Fields. Systemic administration of alternate alkylating agents is less well tolerated than the TMZ and is usually reserved for tumor recurrence (6). There are different reports regarding the effect of bevacizumab on overall survival (7), but it does decrease vasogenic edema and can temporarily improve the quality of life for inoperable recurrent disease resistant to standard therapies. However, the phenomenon of “pseudoresponse” has been well reported after bevacizumab therapy, in which the imaging signs of decreased BBB permeability (and vessel leakage) are not reflective of a true decrease in the tumor burden (8). Despite standard multi-modal therapy, the median survival of patients with HGG remains poor, particularly in the setting of isocitrate dehydrogenase (IDH) wild-type tumors. In the case of GBM, the most aggressive form of HGG which comprises nearly 50% of all gliomas, the median survival is approximately 15 months (5). These poor outcomes have motivated the efforts to identify novel therapeutic strategies, including targeted small molecule therapies, metabolically-targeted therapies, and immunotherapies, in attempts to improve the treatment response and overall survival.

Many emerging passive and active immunotherapy treatments for HGG are currently being evaluated in clinical trials, attempting to combat the immunosuppressive characteristics of glioma to bolster the treatment efficacy (9, 10). The HGGs have been shown to elicit severe reductions in the number and function of CD4 T lymphocytes (11). This can be reversed with the blockade of inhibitory pathways leading to increased T-cell response and activation, and subsequently increased the antitumor activity of these immune cells. Mouse models have demonstrated improved survival with the use of anti-programmed cell death protein (anti-PD-1) immune checkpoint inhibitors for intracranial glioma (12), and nivolumab and pembrolizumab, both anti-PD-1 agents, have been evaluated in multiple recent and ongoing clinical trials. Cytotoxic T-lymphocyte-associated protein 4 (CTLA-4), lymphocyte activation gene 3 (Lag3), and indoleamine 2,3-dioxygenase (IDO) are additional immune checkpoint inhibitors that affect the T- lymphocyte activation pathway, and they have induced tumor regression and improved the long-term survival in murine glioma models, particularly when used in combination (11).

Active immunotherapy regimens capable of inducing antitumor response include multiple vaccine-based treatments. Dendritic cell-based vaccines, already FDA-approved for prostate cancer treatment, also increased survival in preclinical GBM models (13). Dendritic cells are a type of antigen-presenting cell derived from a patient's CD14+ monocytes, loaded with tumor antigens *in vitro*, and subsequently injected back into the patient to activate T-cell and B-cell responses (14). Peptide vaccines are comprised of synthetic amino acid chains mimicking proteins that are over-expressed by tumors. Current peptide vaccines under investigation target the isocitrate dehydrogenase 1 (IDH1), epidermal growth factor receptor variant III (EGFRvIII), and human leukocyte antigens (HLAs) (10). Oncolytic viruses, already FDA-approved for metastatic melanoma, are recombinant viruses, such as adenovirus or herpes simplex virus (HSV) that are used to infect and destroy tumor cells. The safety, efficacy, and long-term effects of oncolytic viruses in glioma treatment are currently under investigation (9). Chimeric antigen receptor (CAR)-T cell therapy utilizes CARs that are bound to a patient's T-cells (15). The CAR-T cell therapy is FDA-approved for hematologic malignancies (9), showing robust efficacy in preclinical models and has demonstrated a proof of principle in refractory human GBM (16). Despite a strong biological premise and promise in the early phase trials, showing evidence of biological efficacy based upon early radiographic evidence of pseudoprogression, no immunotherapy regimen has yet demonstrated improved survival in a randomized phase 3 trial for GBM. These translational challenges increase the urgency to improve individual longitudinal analysis of individual human gliomas through improved radiographic analyses.

## Treatment-Related Effects

Posttreatment radiation effects (PTREs), which include pseudoprogression and radiation necrosis, have been well documented following chemoradiation, with a similar phenomenon following immunotherapy (17, 18), and can confound the assessment of treatment response both clinically and on imaging findings. A spectrum of imaging findings and histologic changes in the treated tumor field can be presented with or without associated clinical symptoms. Pseudoprogression has been defined as treatment effects immediately after or soon after radiotherapy leading to perceived tumor progression on the follow-up imaging and subsequent improvement without any change in the intervention (17). Pseudoprogression is generally used to describe early PTRE occurring within 3–6 months after the initiation of adjuvant therapy (19), while radiation necrosis typically occurs beyond 6 months of completing the therapy, but can occur early, mid, or late (even after many years) after the completion of adjuvant therapy (20). Both entities are believed to be manifestations of radiation-induced effects, and both were documented in postradiotherapy patients prior to the widespread use of TMZ (21). However, the added effect of TMZ appears to increase the incidence of pseudoprogression and radiation necrosis (22, 23), presumably due to increased radiosensitization by the cytotoxic agent (19, 24). Pseudoprogression has been associated with improved overall survival in multiple studies (25, 26), and it has been suggested that the presence of pseudoprogression could correlate with the efficacy of therapy (27). The presence of radiation necrosis has also been associated with the improved rate of survival, although some studies suggest that this association may be influenced by the timing of radiation necrosis development and confirmation on biopsy. One study reported improved survival when radiation necrosis was diagnosed from biopsies obtained more than 5 months after treatment (28). Another study suggests that radiation necrosis is associated with improved progression-free survival as well as the overall survival from the time of recurrence, rather than from the initial diagnosis (29).

The pathophysiology of PTRE is not completely understood but is suggested to be a combination of inflammation, edema, myelin destruction, and changes in the vessel permeability resulting in the breakdown of BBB (30). These findings may be transient in the setting of pseudoprogression but do not always resolve spontaneously with radiation necrosis. Pathologic specimens of pseudoprogression changes have been described as bland necrosis, vascular fibrinoid necrosis, reactive gliosis, demyelination, and vascular hyalinization (31) and there is considerable overlap with the pathologic changes seen with radiation necrosis. The incidence of pseudoprogression has a wide variation across studies, but a meta-analysis of 73 studies demonstrated a pooled incidence of 36% after standard therapy for HGG with a range of 0–64% (32). A greater incidence of pseudoprogression has been shown in gliomas with methylated MGMT promoter status compared to the unmethylated status (27). The reported incidence of radiation necrosis ranges from 3 to 24% (3). Clinical symptoms can accompany the development of PTRE and include headache, nausea, or dizziness that are related to increased intracranial pressure. These symptoms are typically managed with steroid therapy unless the patient is taking immune checkpoint inhibitor therapy, in which case, symptoms are managed with bevacizumab, as steroids counteract the therapeutic effects of immune checkpoint inhibitors. In some cases, additional treatments are required, including bevacizumab, Laser Interstitial Thermal Therapy, or surgical resection (33).

Immunotherapy for gliomas and other cancers is well documented to increase the incidence of pseudoprogression (34, 35). The mechanism is not fully elucidated but is presumably related to the direct inflammatory response caused by the activation of the immune system (36). Reports of histologic changes specific to glioma are lacking, but the histology of pseudoprogression seen in the melanoma of brain metastases demonstrated clusters of tumor cells with surrounding reactive astrocytosis, inflammatory cells, and microglial cells (37). Generalized neuroradiologic changes and neurotoxicities, such as aseptic encephalitis or meningitis, have also been documented in the use of immunotherapy for tumors outside of the CNS (38–40), and these changes could contribute to the posttreatment effects of immunotherapy for HGG.

## Current Use of Posttreatment Imaging Follow-Up

Contrast-enhanced MRI is considered the golden standard for the follow-up of brain tumors, and the protocols are largely based on the standardized brain tumor imaging protocol (BTIP) consensus recommendations published in 2015 (2). The minimum standard protocol includes 3D pre- and postcontrast T1-weighted sequences, axial 2D T2-weighted sequence, axial 2D FLAIR, and axial 2D diffusion-weighted imaging (DWI). Following surgical resection, baseline postoperative imaging is usually acquired within 24–48 h to evaluate the degree of tumor removal, which has important prognostic implications (41), and to provide a baseline for subsequent imaging. The National Comprehensive Cancer Network (NCCN) guidelines recommend an initial follow-up imaging of 2–8 weeks later following the completion of adjuvant therapy (1). Subsequent imaging will be spaced out every 2–4 months for 3 years and then every 3–6 months indefinitely.

Traditionally, response assessment was determined on the basis of measurable enhancement using the Macdonald Criteria (42). The 2010 Response assessment in neuro-oncology (RANO) criteria have since superseded the Macdonald Criteria and account for other imaging characteristics that may be present in addition to or instead of an enhancement (43). In particular, the RANO criteria acknowledge that gliomas may demonstrate T2/FLAIR changes only without enhancement. Therefore, the RANO criteria define tumor progression as “greater than or equal to 25% increase in the sum of the products of perpendicular diameters of enhancing lesions measurable enhancement” or “significant progression in T2/FLAIR changes” (43). These criteria also take into account the changes in steroids and symptoms to help compensate for the limitations of interpreting imaging without clinical context (44). Due to the prevalence of pseudoprogression within the first 3 months of initiating therapy, the RANO criteria recommend defining progression within the first 3 months only if it is confirmed pathologically or if the majority of enhancement is outside the radiation field. Given the multiple emerging immunotherapy options for brain tumors, the immunotherapy RANO (iRANO) criteria were later published in 2015 and further expand on the recommendations related to pseudoprogression (36). In patients with the early imaging signs of progression, within 6 months of initiating immunotherapy, iRANO advocates for confirmatory imaging no sooner than 3 months after the initial radiologic progression. Despite the development of these criteria, there are limitations described below that limit the practical application in the routine clinical use, due in large part to the non-specificity of contrast-enhancement (which could represent tumor or posttreatment effect) and histologic admixture within a single lesion.

### Limitations of Conventional CE-MRI and Challenges Due to Intratumoral Heterogeneity

The overlapping imaging characteristics of PTRE and tumor progression on CE-MRI are well recognized (17, 19, 36, 43). Both tumor and PTRE can present as enlarging, enhancing masses with the surrounding vasogenic edema on serial conventional CE-MRI examinations. These imaging features relate to the extravasation of contrast into the tissues secondary to vascular leakage and BBB disruption (45). The non-enhancing edema seen on T2/FLAIR imaging is presumably related to increased water content from associated vascular leakage. However, this can be further complicated by the likely admixture of non-enhancing tumors that can infiltrate the peritumoral vasogenic edema regions surrounding the enhancing core (46).

Aside from the difficulties in distinguishing the appearance of tumor and PTRE on CE-MRI, histologic admixture commonly occurs, such that tumor growth can (and arguably typically does) occur within a background of treatment-related changes (46–49). This admixture can occur within a single biopsy specimen, as highlighted recently in a study by Winter et al. (50) from a large cohort of biopsy- proven cases of pseudoprogression and radiation necrosis. But at the same time, studies have also shown that foci of tumor recurrence can be spatially distinct from the regions of pure PTRE within different regions of a single MRI-enhancing lesion, which suggests the different spatial scales of intralesional histologic heterogeneity (51, 52). While most studies on intralesional heterogeneity have been within the context of the posttreatment setting following standard chemo-radiation, intralesional heterogeneity is also well documented following immune therapies, with admixed regions of tumor growth co-occurring with the adjacent regions of pseudoprogression/posttreatment changes (36, 53). Importantly, this heterogeneity also increases the risk of surgical sampling errors, as the histologic diagnosis from one biopsy location may not accurately reflect the diagnosis from other regions of the same MRI enhancing lesion (46). Further, variability in the histologic admixture within individual biopsy specimens also opens the door to variability in histologic criteria to define HGG vs. PTRE. The clinical utility of quantifying relative tumor burden within an enhancing lesion is still being investigated, as some studies have shown that histologic tumor fraction (i.e., the proportion of residual tumor) has important prognostic implications (54), while others failed to show a significant correlation with the survival (55). However, to the extent that imaging may be relevant to interpret the therapeutic response to a presumed recurrent tumor, the ability to reliably discriminate tumor from treatment effects could help accelerate the therapeutic translation and help decrease the guesswork currently inherent to patient management.

## Advanced Imaging Techniques and Principles

### Perfusion Imaging

Perfusion MRI comprises a variety of functional imaging techniques, supplemental to conventional MRI, that can provide additional information related to hemodynamics and vascularity of tissues. Perfusion techniques include dynamic susceptibility contrast (DSC), dynamic contrast enhanced (DCE), and arterial spin labeling (ASL) imaging. Both DCE and DSC require exogenous GBCA while ASL uses the endogenous contrast of magnetic labeling. Many studies have shown that perfusion MRI techniques, in general, can provide valuable additional information to standard MRI in the assessment of brain tumors, particularly when assessing the post-treatment effects of HGG.

Dynamic susceptibility contrast-based perfusion imaging is based on the paramagnetic susceptibility effects of GBCA on the acquired MRI signal. DSC is also termed “bolus-tracking” MRI, as the first pass of a contrast bolus through the brain tissue is dynamically evaluated with gradient echo (GRE) T2^*^-weighted or spin echo (SE) T2-weighted images (56, 57). The intravascular and extravascular compartments experience differing susceptibility effects from GBCA and each contributes to the acquired signal. The voxel-wise change in signal over time can be mapped to a contrast concentration-time curve which can be further analyzed to create parametric maps of relative cerebral blood volume (rCBV) and relative cerebral blood flow (rCBF). Additional derived parameters include peak height (PH) and the percentage of signal intensity recovery (PSR). PH is defined as the maximum signal intensity loss during bolus tracking compared to the baseline, and it has been shown to correlate with CBV (58). PSR is based on the signal recovery relative to baseline after the first pass of contrast bolus and is influenced by vessel permeability, acquisition parameters, as well as cell density and cell size (59).

**Figure 1:**
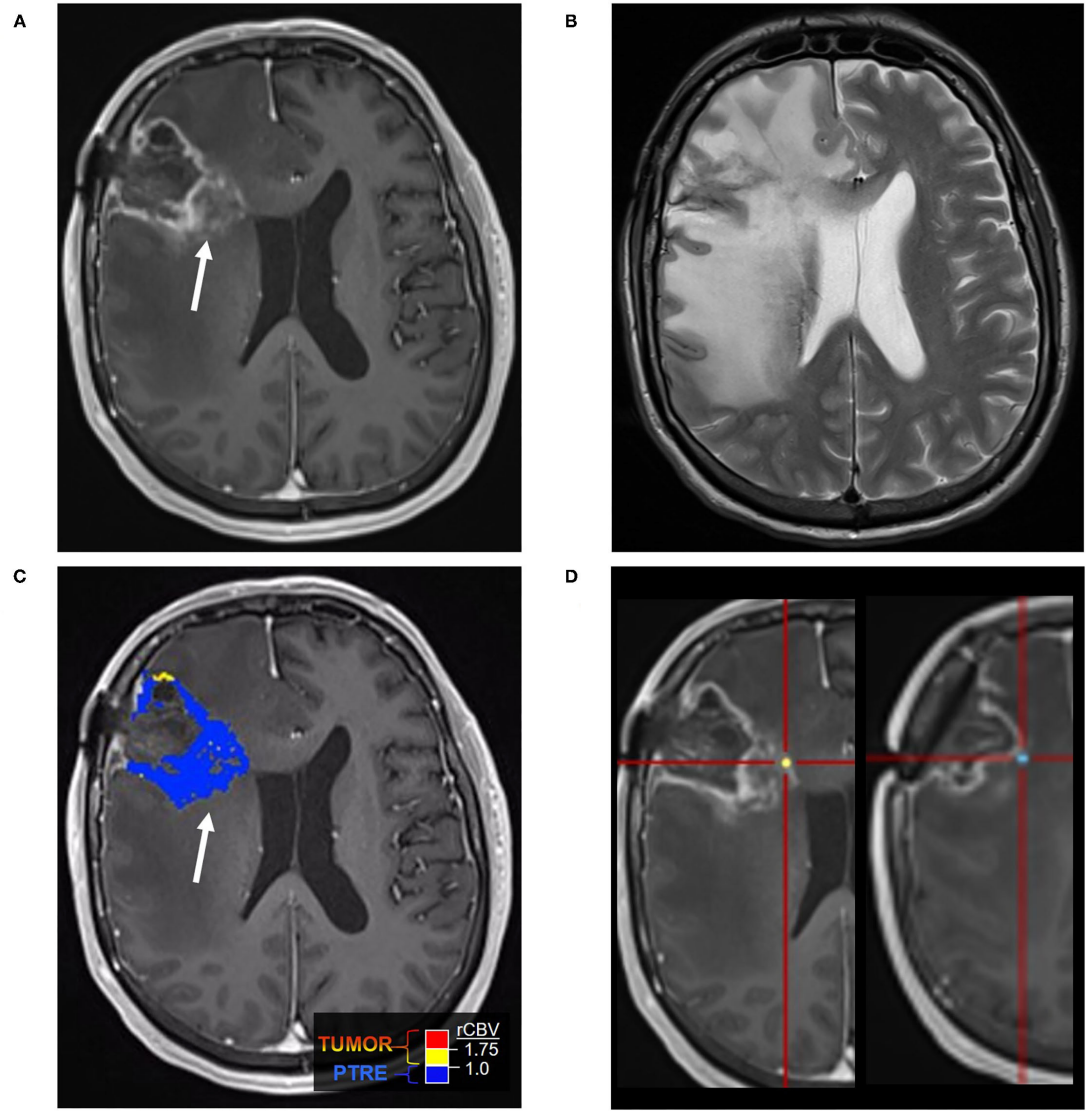
Posttreatment changes on Dynamic Susceptibility (DSC) MRI: A 58-year-old man with right frontal glioblastoma (GBM) status post resection and adjuvant radiation with concomitant temozolomide presented 6 months after resection with left facial droop and left-sided weakness. Initial CT and Contrast-enhanced MRI (CE-MRI) showed an increased mass effect and ill-defined enhancement around the resection cavity, indeterminant for recurrent tumor vs. posttreatment effects. **(A)** Axial postcontrast T1-weighted image demonstrates a linear enhancement around the right frontal resection cavity with more ill-defined nodular enhancement along the posteromedial margin (arrow). **(B)** Axial T2-weighted image shows T2 hyperintense signal and mass effect throughout the right frontal and parietal lobes. **(C)** DSC Fractional tumor burden (FTB) map derived from rCBV thresholds shows low FTB around the resection cavity favoring posttreatment changes **(D)** Stereotactic biopsies were performed and cross-registered retrospectively to areas of enhancement. Pathology demonstrated virtually entirely necrotic tissue with no viable tumor, concordant with the rCBV map.

**Figure 2:**
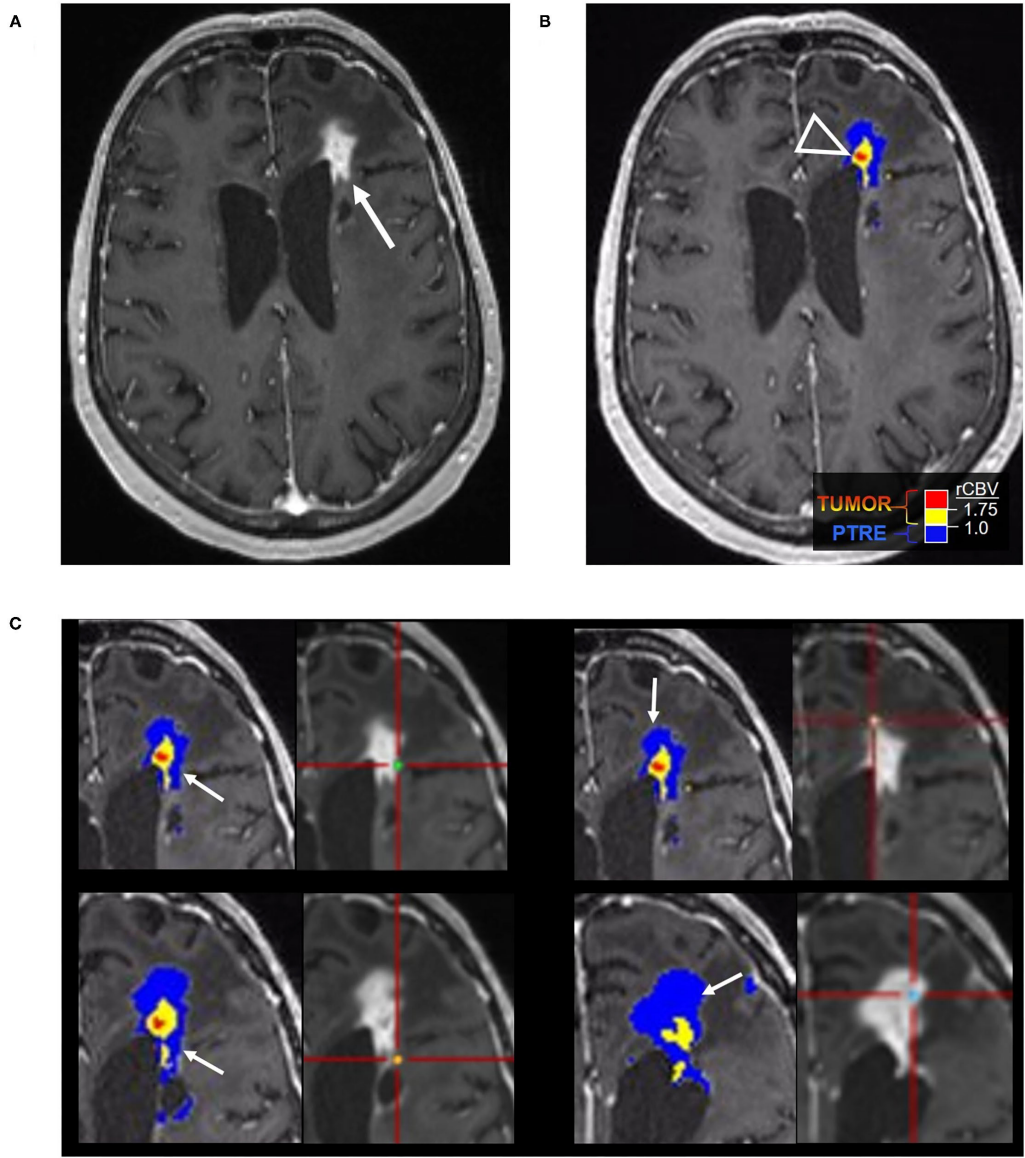
Posttreatment changes on DSC-MRI: A 68-year-old woman with left frontal GBM with two prior surgical resections and adjuvant radiation, temozolomide, and lomustine therapy undergoing routine surveillance imaging. **(A)** CE-MRI 2 years after initial diagnosis demonstrated increased nodular enhancement along the resection cavity concerning recurrent tumor. **(B)** The DSC FTB map derived from relative cerebral blood volume (rCBV) thresholds demonstrate a small area of high FTB centrally (arrowhead) suggesting tumor recurrence, but the majority of the surrounding tissue had low rCBV consistent with posttreatment effect. **(C)** Stereotactic biopsies were performed and cross-registered with areas of enhancement, but only corresponded with low rCBV regions. Pathology showed scant atypical cells in a background of extensive therapy-related changes, compatible with the rCBV map. No viable GBM was identified. Unfortunately, the central portion of the tumor was not sampled and cross-registration with the DSC images at the time of biopsy may have been helpful in fully characterizing the lesion pathologically.

**Figure 3:**
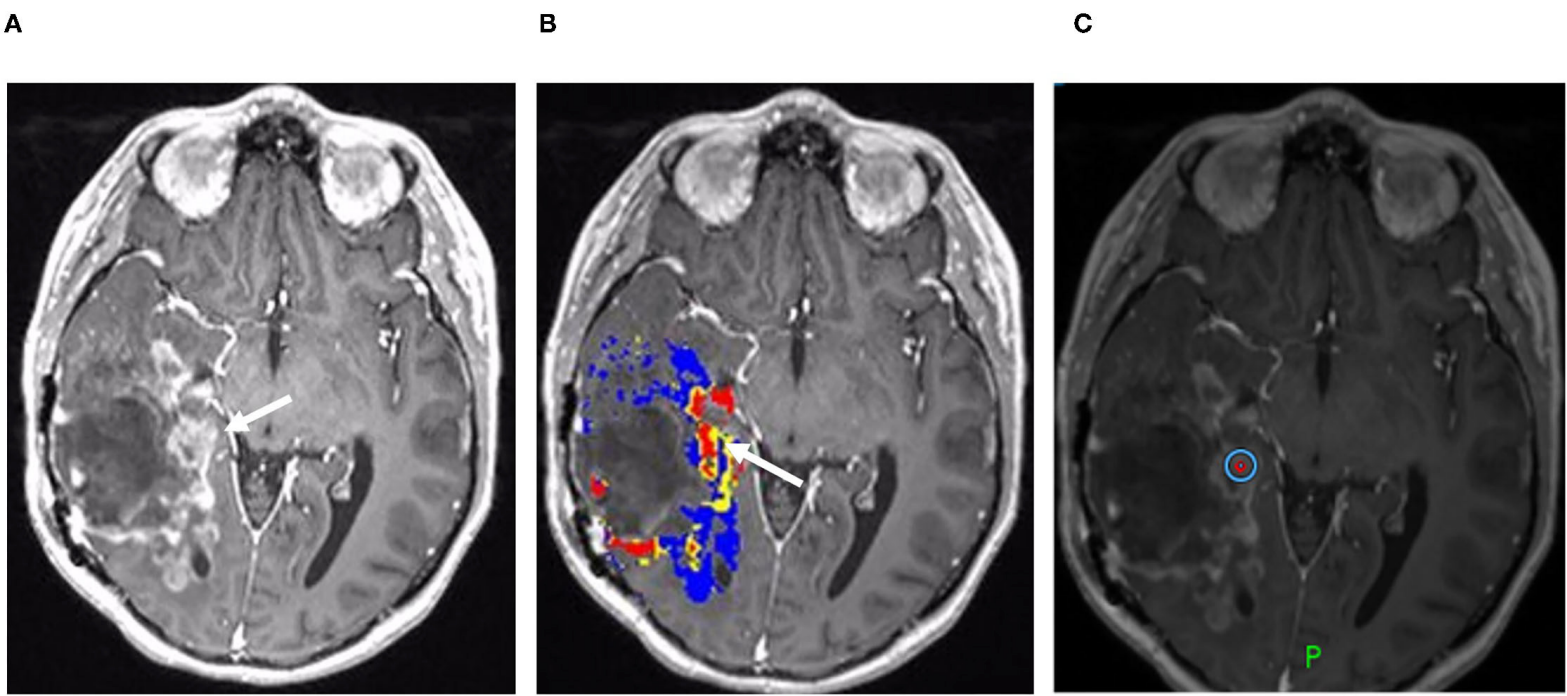
Recurrent tumor on DSC-MRI: A 27-year-old man with right temporoparietal GBM with prior surgical resection with carmustine wafer placement and adjuvant radiation/temozolomide. Five months after surgical resection, the patient experienced clinical deterioration with signs of increased intracranial pressure with clinical concern for tumor progression vs. pseudoprogression. His symptoms did improve with steroid therapy. **(A)** Axial postcontrast T1 weighted image shows a large area of nodular and ring-like enhancement along the medial margin of the resection cavity (arrow). **(B)** The DSC FTB map derived from rCBV thresholds demonstrates high FTB centrally in the area of nodular enhancement consistent with the tumor. **(C)** The patient underwent surgical debulking, and the stereotactic biopsy at the site of the elevated rCBV (circular marker) was positive for 80% recurrent viable tumor.

Among these various advanced DSC-MRI metrics, rCBV represents the most commonly employed and widely published metric for distinguishing tumor recurrence from PTRE. This is based on the biological premise that vascular volume (and thus rCBV measurement) significantly differs between the two entities, as HGG recurrence exhibits higher microvessel volume compared to the PTRE (60, 61) (Figures 1–3). As such, tumor is associated with higher rCBV compared to PTRE. Given the quantitative nature of rCBV, and the clinical importance of distinguishing tumor from PTRE, there has been tremendous interest in identifying reliable rCBV thresholds to differentiate HGG from treatment effects (57, 62–67). A recent meta-analysis by Patel et. al. (68) has highlighted many of these studies and reported 28 studies using DSC-MRI for evaluating gliomas after radiation and/or chemotherapy, a pooled accuracy of approximately 85% for mean rCBV. This body of literature, and the clinical accessibility of the DSC-MRI technique, have helped drive widespread clinical adoption of DSC-MRI for rCBV measurement to aid in the differential diagnosis of tumor vs. PTRE for HGG-response assessment. At the same time, this meta-analysis also highlighted the wide variability in the reported rCBV thresholds across different studies, which ranged from 0.9 to 2.15 for mean rCBV. This variability has likely led to confusion within the neuro-oncologic field regarding the reliability of the predictive diagnostic threshold criteria to distinguish tumors from PTRE. For example, one institution may recommend the rCBV threshold of 1.0 (above which would favor the diagnosis of tumor, and below which would favor the diagnosis of PTRE), while another institution may favor an rCBV threshold of 1.75. While several important factors contribute to this variability, addressing these issues will facilitate the development of universal diagnostic guidelines across institutions for incorporating rCBV thresholds in response assessment.

The first factor relates to the differences in the validation methods that compare rCBV with the histologic classification of tumor vs. PTRE. For example, in the first type of validation method, some studies lack surgical biopsy specimens and histologically confirmed diagnoses when classifying MRI enhancing lesions as tumor vs. PTRE (69, 70). While these studies instead rely on serial imaging, the non-specific nature of MRI, and the fact that PTRE can exactly mimic tumor as enlarging enhancing masses on MRI, may lead to the misclassification of tumor vs. PTRE, which can confound the reliability of the reported rCBV thresholds distinguishing the two entities. Furthermore, as discussed above, the spatial and histologic heterogeneity of tumor and PTRE can complicate the use of CE-MRI in tracking the temporal evolution of enhancing lesions, as the radiographic appearance of MRI enhancement and edema may be influenced by the relative abundance of tumor and PTRE. Meanwhile, the second type of validation method is the use of non-localizing biopsies as a form of histologic confirmation to classify MRI enhancing lesions as tumor vs. PTRE (71). This generally involves the use of a biopsy specimen from an unspecified location within the MRI enhancing lesion to classify the entire lesion as either tumor or PTRE, based on histologic criteria. As the location of the biopsy (and thus the corresponding location for rCBV measurement) is undocumented, the rCBV value of a particular lesion is generally measured from the entire MRI enhancing volume. This creates a spatial discrepancy between the measured rCBV and the histologic diagnosis, which can be confounded by intratumoral heterogeneity (as detailed in the previous heading), which increases the risk of sampling errors in the diagnosis and histologic misclassification (Figure 2). Also, as MRI enhancing lesions invariably comprise a variable histologic admixture of tumor and PTRE components, the measured rCBV across the entire lesion is likely influenced by these admixed components. Finally, there are also differences in the histologic criteria across studies that are used to define tumor vs. PTRE classifications. For instance, one study required that surgical samples contain at least 20% of tumor components to classify an MRI enhancing lesion in the “tumor” category (64), while lower histologic thresholds (i.e., 0–20%) were used by other studies (49, 51, 52, 61, 72). All of these factors can not only influence the accuracy of the reported rCBV thresholds to distinguish tumor from PTRE but also likely contribute to the variability in the reported threshold values themselves, as published by one institution vs. another. The third type of validation method relates to the use of image-localized biopsies to record the stereotactic locations of surgical specimens that are submitted for histologic diagnosis, which are then spatially correlated with the localization of regional rCBV measurements from the corresponding biopsy locations. There are several distinct advantages to this method of histologic validation. This spatially accurate method of correlating the localized rCBV with individual biopsy samples addresses the confounds of histologic heterogeneity at the regional spatial scale, which can refine the rCBV thresholds distinguishing tumors from PTRE. Also, the use of individual biopsy specimens as separate “observations” within a large enhancing mass can improve the likelihood of isolating histologically “pure” PTRE vs. “pure” tumor (49, 52, 61), which addresses the histologic heterogeneity at the biopsy scale. Finally, the use of localized regional rCBV measurements (on the order of less than 10 voxels per region of interest) can facilitate the identification of rCBV thresholds that distinguish tumor vs. PTRE at the regional level, and even at the voxel level, which allows for the use of rCBV to differentiate and quantify the regions of tumor vs. PTRE within the admixed lesions (46, 49, 51, 52, 61, 73). Identifying regionally-specific tumor populations and the regions of PTRE can help overcome clinical sampling errors by guiding surgical biopsy targets that are specific to foci of tumor recurrence which not only aids clinical diagnostic confirmation of tumor progression but can also help isolate adequate tumor burden for other applications, such as molecular profiling and investigational assays.

A second factor contributing to the variability in rCBV thresholds across published studies relates to differences in the acquisition methods for clinical DSC-MRI. The meta-analysis by Patel et al. (68) highlighted the broad variability in pulse sequence parameters, principally the repetition time (TR), echo time (TE), flip angle (FA), and contrast agent dosing. Variations in these pulse sequence parameters can affect the measurement of rCBV, particularly in the setting of BBB disruption with resultant contrast extravasation, which can result in the so-called “T1W leakage effects” that lead to underestimates of rCBV (74). While T1W leakage effects are exaggerated by specific pulse sequence parameters (e.g., higher FA, a spin-echo technique), a number of studies have revealed techniques to minimize their influence. For instance, the use of preload bolus injection was introduced to “pre-saturate” T1W shortening prior to a second bolus injection for DSC-MRI acquisition (75, 76). A full-dose preload of 0.1 mmol/kg and the incubation time of 6 min in combination was found to optimize the differentiation of rCBVs between true tumor progression and PTREs, when validated against coregistered stereotactic biopsy specimens (77). The body of literature regarding the DSC-MRI accuracy has motivated the development and publication of consensus recommendations for DSC-MRI acquisition (57). This stemmed from initial efforts from the American Society of Functional Neuroradiology (ASFNR), which released DSC protocol recommendations in 2015 prior to the BTIP consensus recommendations (78). Building upon the ASFNR guidelines, the Jumpstarting Brain Tumor Drug Development Coalition Imaging Standardization Committee subsequently published the updated consensus recommendations for the DSC MRI protocol in 2020 (57). The consensus guidelines address the multiple protocol decisions that must be addressed for reliable CBV measurements. In addressing acquisition parameters, GRE sequences are recommended over spin echo, as the T2^*^W effects of GRE are more sensitive to the larger vessels present in the gliomas. The GRE DSC effect is also proportional to GBCA concentration over a broader range of vessel sizes, and GRE is less sensitive to changes in tissue-diffusion coefficient, which leads to greater rCBV accuracy. The recommended acquisition parameters, including the TR, TE, and FA, derived from computational studies aimed to optimize rCBV accuracy (79). The selected parameters were subsequently validated in patients. These parameters are summarized in Table 1.

**Table 1:** Consensus Guidelines for DSC MRI by Boxerman et al. (57).

**Pulse sequence**	**Gradient echo**
Mode	2D
Dosing protocol and flip angle (FA)	Pre-load + Bolus: 30° or 60° FA
	Bolus only: 30° FA
Echo time	3T: 30 ms (25–35 ms for 30° FA or 20–25 ms for 60° FA)
	1.5T: 45 ms (40–50 ms)
Repetition time	1,000–1,500 ms
Total time points	≥120
Baseline time points	50 (30–50)

A third factor contributing to variability in the published rCBV thresholds relates to the use of postprocessing model-based correction for rCBV measurements. Model-based postprocessing is required to calculate “corrected rCBV” values that account for both T1W leakage effects and T2^*^W residual effects that may persist despite optimal DSC acquisition conditions (74, 77). A model-based correction has been shown necessary, in addition to the use of specific pulse sequence acquisition parameters, to fully correct for T1W leakage effects (as well as confounding residual T2^*^W^*^ effects) (57). While there are a wide variety of model-based correction methods, some of the commercially available studies have shown that not all software methods can generate reliable corrected rCBV values. At least, 16 different software packages were used across the 28 studies of Patel et al.'s (68) meta-analysis. Different software modeling and postprocessing algorithms impact the accuracy and reproducibility of perfusion imaging. For instance, Conte et al. (80) studied the intraobserver and interobserver variability of DSC and DCE interpretation of 20 patients with two different commercial software packages and different postprocessing choices. Good intraobserver and interobserver reproducibility was seen with the use of particular software, but only “fair to moderate” agreement was seen with different software applications (80). Hu et al. (81) evaluated leakage correction and the rCBV calculations of 52 patients using two different commercial software packages (IB Neuro from Imaging Biometrics and nordicICE from NordicNeuroLab). In 12 of the patients, the parameters were also validated with histologic benchmarks from stereotactic biopsies. The study concluded that the IB Neuro software was more accurate in diagnosing tumor vs. posttreatment effects. This has motivated efforts by the quantitative imaging network (QIN) to compare broader arrays of model-based correction methods to work toward consensus recommendations (82–84).

#### Recommendations on rCBV Thresholds to Distinguish Tumor From PTRE Following Standard Chemo-Radiation Therapy

Based on the issues discussed above, we recommend that the selection of a numerical rCBV threshold, to clinically distinguish tumor from PTRE, should be based on those reported thresholds that have been spatially validated through the use of image-localized biopsies. Due to the regionally specific nature of this method of validation, the rCBV thresholds are more appropriately applied to distinguish tumor vs. PTRE at the regional voxel level, rather than for a global single mean value across an entire enhancing MRI lesion (49, 52). The threshold of 1.0 normalized rCBV has been validated by Hu et al. (49) and provides a straightforward guideline for differentiating tumor (rCBV > 1.0) from PTRE (rCBV ≤ 1.0). This value is derived by “normalizing” rCBV against CBV from two regions of interest (ROIs), placed within the normal appearing white matter (NAWM) adjacent to the frontal horn, and also within the corona radiata in the deep frontoparietal region, as seen in the Supplemental On-Line Figure 1 by Hoxworth et al. (51). An early study by Hu et al. (61) reported a lower threshold (rCBV = 0.71) by normalizing the CBV against both the NAWM and deep gray matter structures in the basal ganglia. As the gray matter structures can exhibit higher microvessel density compared to the white matter, using the gray matter to normalize CBV will by definition result in lower normalized rCBV values. A separate study by Hu et al. (77) found that NAWM demonstrated greater stability in CBV over multiple preload dose injections, compared to the normal gray matter structures. As a result, our recommendation is to follow the simpler approach of using NAWM to normalize CBV and to use the rCBV threshold of 1.0. While this threshold was validated for distinguishing the biopsy samples with any amount of tumor (i.e., greater than 0% tumor) from biopsy samples with pure PTRE (i.e., 0% tumor), Prah et al. (52) performed a separate image-localized biopsy study and validated a similar rCBV threshold to distinguish the pure tumor (i.e., 100% tumor) from pure PTRE (i.e., 0% tumor) biopsy specimens. Of note, these rCBV thresholds are specific to postprocessing software (49, 51, 52, 81), and the thresholds may need to be modified if other postprocessing software methods are used. As an alternative to the normalization process, which requires the user-input to place NAWM ROIs, the use of standardized rCBV (std-rCBV) values has been proposed to help offset the potential variabilities in user-defined inputs (85). A std-rCBV threshold to distinguish tumor from PTRE was first validated by Prah et al. (52), employing the image-localized biopsies. The scale of the std-rCBV reported by Prah et al. (52) (3,575 a.u.) has since been modified to mirror the scale of normalized rCBV values (1.0 a.u), and the threshold of 1.0 for std-rCBV was validated subsequently by Hoxworth et al. (51) using image-localized biopsies to reliably distinguish HGG recurrence PTRE. As such, our recommendation of the threshold of 1.0 can be used for both normalized rCBV and standardized rCBV measurements to distinguish the HGG recurrence from PTRE. This can be applied to measurable enhancing lesions both within and beyond the radiation field. Of note, these thresholds have not yet been validated for other tumor types, such as metastases or primary CNS lymphoma. Application of these thresholds for non-measurable disease (defined by RANO criteria) has also not been validated, particularly for non-enhancing T2/FLAIR regions, and should represent an important focus for future studies. With respect to non-measurable enhancing lesions, care should be taken not to “over-interpret” the rCBV maps in setting the small lesions (on the order of several millimeters), as the DSC-MRI technique has an inherent limitation to spatial resolution (voxel sizes in the order of several millimeters).

#### Use of RCBV in the Setting of Anti-angiogenic Therapy

Anti-angiogenic therapies like bevacizumab demonstrate a strong effect on vessel permeability, which decreases as a result of the blockade in VEGF pathways. Post-processing leakage correction algorithms are particularly important in this setting, to offset potential technical influences of rCBV measurement that result from alterations in T1W leakage effects across serial examinations. Anti-angiogenic therapies also likely exert a measurable decrease in the microvessel volume, which has been shown in preclinical models to be due in part to pericyte contraction (86). Clinical studies utilizing dual-echo GE/SE DSC MRI before, during, and after anti-angiogenic therapy have shown decreased relative vessel size, particularly affecting large-caliber microvessels (those greater than 5–10 microns in diameter) (87). Despite these observed decreases in tumoral rCBV following anti-angiogenic therapy, longitudinal clinical trial data have shown that the rCBV values themselves, in many cases, do not necessarily fall below the spatially-validated thresholds for distinguishing tumor from PTRE (88), including those for standardized rCBV (51, 52) and normalized rCBV (49). For example, if we were to consider the standardized rCBV threshold (greater than 3,575 a.u. represents tumor, less than 3,575 represents PTRE), as reported by Prah et al. (52) that was spatially validated using image-localized biopsies, then we observe that in **Figure 5A** of the study by Schmainda et al. (88), many of the lesions with elevated rCBV above the threshold (presumably representing tumor) on the pretreatment timepoint (TP1), continued to retain the elevated rCBV values above the threshold at the posttreatment time point (TP2). As such, despite a serial decrease in rCBV following anti-angiogenic therapy, an absolute rCBV value that remains above the threshold would still provide value in distinguishing HGG recurrence from PTRE. In contrast, for those lesions that demonstrated a fall in rCBV below the threshold following anti-angiogenic therapy, the rCBV would be non-specific for distinguishing the tumor (with diminished microvessel volume) from PTRE.

#### Utility of DSC-MRI and RCBV to Distinguish Tumor From Pseudoprogression Following Immune Therapies

There have been preliminary studies evaluating the potential prognostic associations between rCBV and RANO-based classification of tumor progression vs. pseudoprogression in HGG following immune-based therapies (34, 89, 90). For instance, Cuccarini et al. (89) analyzed serial rCBV measurements following Dendritic Cell Immunotherapy and found that the serial increase in rCBV was correlated with the likelihood of tumor progression based on RANO criteria. However, similar to standard chemoradiation therapy, immune-based therapies can induce treatment-related changes that result in variable histologic admixture between pseudoprogression and tumor recurrence, with the possibility of a single MRI enhancing lesion demonstrating both the components of PTRE (that resolve over time) as well as components of tumor progression (that increase over time) (36, 53). This presents similar confounding limitations in RANO-based criteria as a surrogate for histologic identity and underscores the critical importance of establishing rCBV guidelines based on histologic validation from surgical tissue specimens using image-localized biopsies. To date, no published studies have validated the use of DSC-MRI metrics, including rCBV, to distinguish HGG recurrence from posttreatment-related effects following immune therapies. As such, no reliable thresholds can be recommended at this time. However, given the increasing use of immune-based therapies in the treatment of HGG, it is paramount to perform such histologic validation studies as a future goal in neuro-oncologic research. For such future studies, it would be important to adhere to consensus recommendations for DSC-MRI acquisition (57).

A potential alternative to gadolinium-based DSC-MRI uses ferumoxytol, an ultrasmall superparamagnetic iron oxide nanoparticle, which has been introduced as an off-label MRI contrast agent and does not extravasate due to its larger particle size (91–94). This technique has been used to distinguish pseudoprogression from tumor recurrence, and, with delayed imaging, provides additional information about neuro-inflammation, as it is phagocytosed by inflammatory cells and potentially localizing cells, such as tumor-associated macrophages (95, 96). However, it is likely to be used in complement to gadolinium-based DSC MRI, as this contrast agent serves as the current clinical standard for CE-MRI in neuro-oncologic practice using the BTIP protocol. Also, there are potential issues, such as the time needed for the clearance of ferumoxytol, as MR image contrast may be altered by ferumoxytol for days to months after administration and by questions related to its safety profile (97).

#### Utility of PSR and PH in Distinguishing HGG Recurrence From PTRE

**Figure 4:**
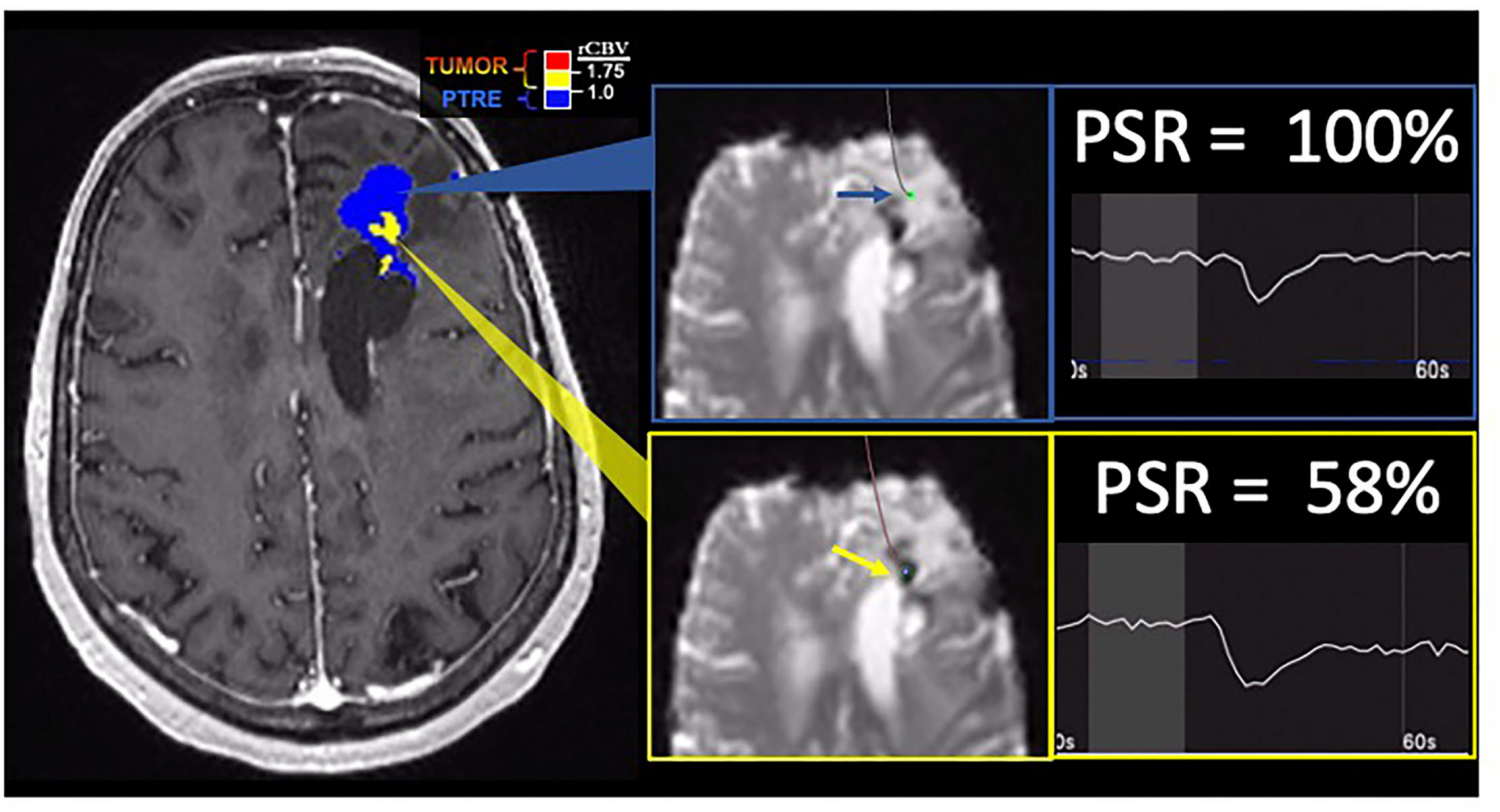
Percentage signal recovery in PTRE and recurrent tumor: A 68-year-old woman with left frontal GBM with two prior surgical resections and adjuvant radiation, temozolomide, and lomustine therapy undergoing routine surveillance imaging. DSC was performed due to concern for tumor recurrence (refer to Figure 2). The FTB map shows peripheral low rCBV (blue) consistent with PTRE which was confirmed on stereotactic biopsy. The central component with higher rCBV (yellow) was presumed to have a recurrent tumor and resected, but not confirmed with stereotactic biopsy. Correlating PSR maps and signal intensity-time curves are provided to demonstrate a high PSR of essentially 100% for the peripheral region consistent with the PTRE and a lower PSR of 58% for the central region that was presumed to have a recurrent tumor.

Young et al. (66) evaluated a cohort of 20 patients with GBM, 9 of whom had pathologic confirmation, although not with spatially resolved image-localized biopsies. In the absence of pathologic specimens, they relied on imaging follow-up and RANO criteria to classify lesions as either tumor progression or as pseudoprogression. None of the pseudoprogression cases were confirmed by biopsy. Their DSC-MRI protocol employed a pulse sequence with the FA of 60 degrees, with no preload dose administration. They found that rCBV was significantly higher in the tumor progression group and that PSR was significantly lower in the tumor progression compared to the pseudoprogression group. They observed similar predictive accuracy when comparing the use of rCBV vs. PSR. In a larger cohort of 57 recurrent patients with GBM, Barajas et al. (72) also evaluated the predictive performance of rCBV, PH, and PSR derived from DSC-MRI (FA = 35 degrees, no preload dose). They were able to confirm PTRE in a larger number of patients (15 out of 17 cases) through validation by surgical biopsy specimens, although these were not spatially resolved using image-localized biopsies. They found that PH, like rCBV, was significantly increased in the tumor compared to the PTRE cases. Similar to the findings of Young et al. (66), they also found that PSR was significantly lower in tumor progression compared to higher PSR values in the PTRE group and that rCBV, PH, and PSR provided similar predictive performance in distinguishing tumors from PTRE (72). Prah et al. (52) compared PSR measurements in spatially localized biopsy specimens that confirmed regional diagnosis of tumor vs. PTRE. They found that rCBV outperformed PSR measurements and that PSR could not reliably distinguish tumor from PTRE. However, this study employed preload dose prior to PSR measurement, which has been shown to degrade the reliability of PSR as a predictive metric (98). As such, future studies employing image-localized biopsy specimens to validate PSR measurements should utilize DSC-MRI acquisitions in the absence of preload dose. An example case of PSR measurement is provided in Figure 4.

#### Dynamic Contrast Enhanced Perfusion Imaging

DCE-MRI is based on the T1 relativity effects of gadolinium in contrast to the T1-weighted imaging before, during, and after contrast administration. The derived signal intensity-time curve depicts the influx of contrast agent into the local vasculature and its extravasation into the extravascular space (56). In brain tumors, K^trans^ is a derived volume transfer constant that primarily reflects capillary permeability by measuring the transfer rate and accumulation of contrast in the extravascular extracellular space (EES). Additional derived parameters include v_e_, a measure of the EES volume fraction, and v_p_, the blood plasma volume fraction. Compared to DSC, fewer studies have evaluated DCE imaging for glioma follow-up, but K^trans^ is the most commonly reported parameter to differentiate tumor response from posttreatment effects (99–101). The 11 DCE studies that were included in the meta-analysis by Patel et al. (68) demonstrated a pooled sensitivity of 89% and specificity of 85%. Reported K^trans^ thresholds have a wide range (0.05– 0.347) (99, 100), and this variability is caused by the same factors for variability discussed previously for DSC studies, including different quantitative models employed in the different certified software vendors. Furthermore, substantial inter-reader variability and lack of reproducibility have been reported with the use of DCE and K^trans^ (102).

Advantages of DCE (relative to DSC) include higher signal-to-noise ratio (SNR), spatial resolution, and reduced sensitivity to susceptibility artifacts (56). There are also fewer contamination effects from adjacent cortical/sulcal vessels, the calvarium, and the paranasal sinuses, which can be helpful in assessing the tumors near the cortical regions and the skull base. The complementary role of DCE in the case of hemosiderin deposition, which can cause artifacts on DSC, also supports the potential utility of using both DCE and DSC in combination. Anzalone et al. (103) reported findings from a multi-center trial comparing DCE and DSC in the setting of pretreatment glioma grading and reported similar performance between the two techniques. However, the clinical translation of DCE has been hindered by several disadvantages, including longer acquisition time, fewer commercially available software packages, and far fewer validation studies comparing DCE metrics in the classification of tumor vs. PTRE. Additionally, DCE assessment of tumors is limited primarily due to the contrast-enhancing components (due to the dependency on BBB leakage). As such, the DCE shows limited applicability in assessing the non-enhancing components of tumors (compared to DSC-MRI). Finally, since DCE methods are less studied compared to the DSC, consensus efforts for standardization of acquisition/postprocessing are less developed. Future studies employing the histologic validation of DCE metrics, to differentiate tumor from PTRE, are needed, particularly taking into account the spatial heterogeneity through the use of image-localized biopsies.

#### Arterial Spin Labeling

ASL represents an alternative perfusion imaging method that does not require exogenous gadolinium contrast agent administration. Tissue perfusion is measured by magnetically labeling the protons in the arterial blood using a 180-degree radiofrequency inversion pulse on blood before it reaches the brain (104). The inversion pulse results in the magnetization of the water molecules in the blood which serve as an endogenous tracer that will alter the magnetization characteristics of the tissue of interest. The ASL data proportional to the cerebral blood flow is derived by subtracting the labeled images at the ROI from the control images prior to magnetic labeling. In evaluating brain tumors, cerebral blood flow (CBF) or tumor blood flow (TBF) is the most commonly reported parameter (105). In a study by Nyberg et al. (106), the authors reported a positive correlation between CBF from a region of interest across the entire MRI enhancing lesions and the histologic quantification of tumor (vs. PTRE elements) taken from a surgical resection material. While the ASL provides an alternative perfusion technique for patients when GBCA is contraindicated or not preferred (e.g., nephrogenic systemic fibrosis and pediatric populations), its widespread use has been limited by disadvantages, such as lower SNR, which increases the difficulty of clinical interpretation. Also, the vast majority of patients with HGG receive GBCA for routine CE-MRI examinations, which allows for DSC-MRI acquisition. Overall, there have been far fewer studies (compared to DSC-MRI) correlating ASL with the differentiation of tumor vs. PTRE (105–108). And to date, no studies have employed image-localized biopsies to directly compare the regional ASL measurements with spatially resolved histopathologic validation to address the confounds of intratumoral heterogeneity. While the ASL measures of CBF do not suffer from contrast agent leakage effects, they can be confounded by transit time delays (time between the tagging and acquisition of pulses) in tumors with highly tortuous vessels, and it is likely that CBF does not provide additional information above and beyond DSC-MRI measures of rCBV, particularly when appropriate acquisition and leakage correction methods are employed. As such, it is likely that the ASL continues to have a limited role for ASL in the routine clinical practice in the context of HGG response assessment.

### Metabolic MRI

Proton (^1^H) MRS is a method of metabolic assessment using the principle that different molecules resonate at slightly different frequencies and thus obtain different signal echoes. Given the significantly high relative ratio of water in the brain, water suppression must be performed with inversion recovery or chemical-shift selective techniques to provide resolution for the detection of these metabolites. These techniques require large volumes of interest for adequate SNR (43). Measurable metabolites include lactate, lipids, choline, glutamate, and 2-hydroxyglutarate. Choline (Cho), often present in cell membrane compounds, is associated with high cell turnover and tumor growth. Lactate can indicate tissue necrosis but is also present in tumor microenvironments by cells undergoing aerobic glycolysis (109). MRS has been shown to correlate with histologic findings in the initial workup of untreated disease, including identifying the molecular signatures of IDH mutations with increased levels of 2-hydroxyglutarate (110) and defining the tumor boundaries (111).

**Figure 5:**
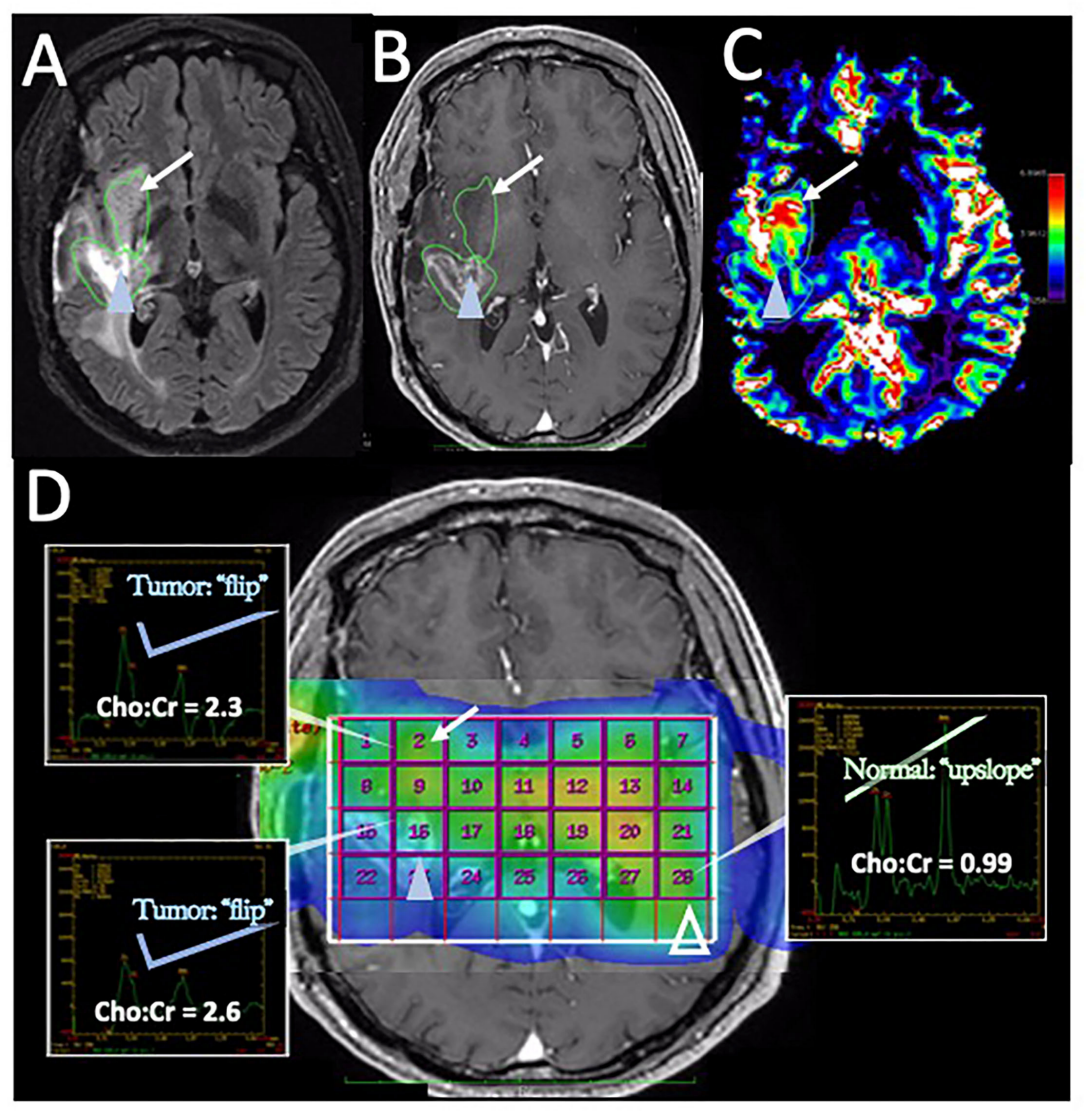
Disease recurrence on DSC-MRI and MRS: A 50-year-old man with right temporal WHO grade III anaplastic oligodendroglioma status post three surgical resections, radiation therapy, and chemotherapy with temozolomide, procarbazine, and lomustine was found to have signs of recurrence on surveillance CE-MRI. **(A)** Axial FLAIR image superior to the resection cavity shows prominent increased FLAIR signal posteriorly (blue arrowhead) and less intense, but more rounded FLAIR signal anteriorly (arrow). **(B)** Axial post-contrast T1-weighted image demonstrates heterogenous enhancement of the posterior region (blue arrowhead) with no significant enhancement in the anterior region (arrow) **(C)** The DSC rCBV map shows increased perfusion of the anterior nonenhancing/FLAIR hyperintense region (arrow) but heterogeneous areas of both increased and decreased perfusion in the enhancing posterior region (arrowhead). **(D)** Magnetic resonance spectroscopy (MRS) demonstrates elevated Cho:Cr ratios in both the anterior nonenhancing (arrow, ratio = 2.3) and the posterior enhancing (solid blue arrowhead, ratio = 2.6) regions. Normal ratios are shown on the contralateral brain (open arrowhead, Cho:Cr ratio = 0.99) for comparison, showing the normal upslope vs. the “Cho-to-Cr flip” seen with the tumor. Both sites with elevated Cho:Cr ratio was concerning for disease progression. The heterogeneous rCBV of the posterior enhancing region was attributed to a combination of disease recurrence and necrotic change. The patient was treated with repeat proton-beam radiation and temozolomide with improvement in the enhancement and FLAIR signal abnormality on subsequent MRI (not shown).

In posttreatment imaging, MRS has shown reasonable accuracy in distinguishing pure tumor or pure necrosis but has limited accuracy in the setting of admixed histology (112). In a well-designed study by Rock et al. (112) employing image-localized biopsies and co-registered MRS measurements in a cohort of 27 recurrent HGG patients, it was found that a Cho to Creatine (Cr) ratio of 1.79 or greater provided 7 times the odds ratio of a biopsy sample yielding pure tumor (compared to pure PTRE). Unfortunately, no values of the MRS ratio could distinguish mixed specimens (containing both tumor and PTRE) in a statistically significant way from either pure tumor or pure PTRE. In a subsequent follow-up study from the same group, Rock et al. (113) reported a further refined Cho-to-Cr ratio threshold of 2.23 that could discriminate the pure tumor from pure PTRE with an odds ratio of 13.56. However, they again found that no MRS ratios could reliably distinguish the admixed specimens from a pure tumor or pure PTRE. Given that histologic heterogeneity commonly occurs in the setting of posttreatment HGG, and the need for large voxel volumes to maximize SNR ratio and the quality of MRS images, the MRS technique has suffered from declining clinical use. MRS also has the disadvantages of technically challenging imaging acquisitions, due to the need to avoid specific anatomic structures (i.e., ventricles, resection cavities, and scalp fat). As such, the scan time can be long if multiple repeated attempts for image acquisition are needed during a clinical examination. While multi-voxel MRS techniques have shown promise in differentiating the treatment recurrence vs. pseudoprogression, (114) this can further reduce SNR and further degrade the image quality, making the clinical interpretation difficult. As such, given the requirements of voxel size, the technical challenges of image acquisition, and the difficulty in assessing the intratumoral heterogeneity, the use of MRS is likely to remain secondary to techniques such as DSC-MRI. Nonetheless, metabolic information can potentially be complementary for specific clinical scenarios, such as the assessment of non-enhancing tumor regions (Figure 5).

#### Machine Learning (ML), Deep Learning (DL), and Advanced Processing Methods for Distinguishing the Tumor Recurrence From PTRE

The field of radiomics has seen extensive growth in the past decade in medical imaging and typically employs a combination of image feature extraction (often through texture analysis) and ML/DL based approaches to develop predictive models for the characterization of diseases, such as glioma (46, 115). Most published studies have focused on the characterization of brain tumors prior to treatment, for instance, to predict the molecular subtypes or disease extent (116, 117). While a detailed discussion of the radiomics methods is beyond the scope of this review, which focuses primarily on perfusion and metabolic MRI, prior studies have applied these methods to help distinguish the tumor from PTRE in the posttreatment setting (118, 119). Other advanced postprocessing methods, such as the use of delayed contrast extravasation, have also been reported to help differentiate the lesions classified as tumor progression vs. pseudoprogression (120). In each of these circumstances, future studies are needed for validation, specifically with the use of spatially resolved image-localized biopsies to help overcome the intratumoral heterogeneity and histologic admixture which can confound a diagnostic performance.

## Summary and Future Directions

Of the advanced imaging methods discussed, the DSC-MRI technique, specifically the measurement of rCBV, provides the most robust and widespread clinically adopted method for response assessment in HGG. For a summary the advanced MRI techniques discussed in this review, please refer to Table 2. This is due in large part to the myriad of published studies consistently demonstrating the high accuracy of rCBV to distinguish the tumor recurrence from PTRE, which represents a persisting clinical challenge for routine CE-MRI. With that said, there remains a need to develop consensus criteria for rCBV thresholds that will guide prospective clinical diagnosis of tumor vs. non-tumoral PTRE. There are several necessary gaps that will need to be addressed to facilitate the development of these diagnostic criteria. One important step has already been addressed, which is the development and publication of consensus recommendations on DSC-MRI acquisition methods (57). One immediate next step will necessitate multi-institutional clinical trials comparing rCBV measurements across clinical sites employing the consensus of DSC-MRI protocols. There is also a need for the development and validation of evidence-based consensus recommendations on model-based correction methods for the calculation of rCBV values, particularly in the context of correcting for T1W leakage effects (and residual T2^*^W effects). This will need to be driven by clinical validation studies employing spatially resolved histologic benchmarks (i.e., image-localized biopsy confirmation of tumor vs. PTRE). In this context, it will be critical to rely on validation techniques, namely image-localized biopsies, to provide spatially resolved datasets of localized rCBV measurements against spatially correlated histologic diagnosis. Given these spatially matched datasets, different leakage correction algorithms can be compared methodologically against one another to support consensus studies on model-based methods, as well as the corresponding rCBV threshold criteria (that may be specific to model-based correction methods) to guide prospective clinical diagnosis. Finally, there remains the need for further studies to understand the potential utility in quantifying tumor burden relative to PTRE in those lesions with histologic admixture. This may help refine clinical diagnostic criteria for response assessment and prognostication and to provide quantitative methods to assess the efficacy of future novel therapeutic strategies.

**Table 2:** Summary of advanced MRI techniques and recommended thresholds for clinical use.

**Imaging technique**	**Imaging metric**	**Tumor**	**PTRE**	**Premise**	**Limitations of technique**	**Reported thresholds from validation studies employing image-localized biopsies**					
						**Threshold**	**Sensitvity**	**Specificity**	**Accuracy**	**Study**	**Notes**
**Summary of advanced MRI techniques**											
DSC-MRI	Normalized rCBV	High	Low	Microvessel volume	Susceptbility from blood products, large cortical vessels can obscure superficial regions	0.71	91.70%	100%	95.90%	Hu et al. (61)	Lower threshold results from normalizing to both NAWM and NAGM; tumor samples could be admixed
						1	100%	100%	100%	Hu et al. (49)	Normalized using only NAWM; only GBM specimens; tumor samples could be admixed
						1.13	82.10%	90%	86%	Prah et al. (52)	Pure GBM vs. pure PTRE; normalized using only NAWM
	Standardized rCBV	High	Low	Microvessel volume		3,575	79.40%	90%	84.70%	Prah et al. (52)	Pure GBM vs. pure PTRE
	Normalized cerebral blood flow	High	Low	Blood flow		1.05	79.40%	80%	79.70%	Prah et al. (52)	Pure GBM vs. pure PTRE
	PSR	Low	High	Leakage, contrast re-circulation		NA		NA = not available, as validation studies employing image-localized biopsies have not been published			
	Peak height	High	Low	Microvessel volume		NA		NA = not available, as validation studies employing image-localized biopsies have not been published			
DCE-MRI	k-trans	High	Low	Vessel leakage	Longer acquisition time, fewer commercially available software packages, limited applicability for evaluating non-enhancing tumor	NA		NA = not available, as validation studies employing image-localized biopsies have not been published			
	vp (plasma volume)	High	Low	Microvessel volume		NA		NA = not available, as validation studies employing image-localized biopsies have not been published			
ASL	Cerebral blood flow	High	Low	Blood flow	May suffer from noisy maps due to limitations in SNR	NA					
						Threshold	Odds ratio pure tumor (vs. pure PTRE)			Study	Notes
MRS	Cho:Cr ratio	High	Low	High cellular membrane turnover	Large voxel volumes required to maximize SNR, limited ability to evaluate regions of histologic admixture between tumor and PTRE; technical challenges in image acquisition	1.79	7			Rock et al. (112)	Pure tumor vs. pure PTRE
						2.23	13.56			Rock et al. (113)	pure tumor vs. pure PTRE

## Author Contributions

All authors listed have made a substantial, direct, and intellectual contribution to the work and approved it for publication.

## Funding

U01 CA250481, U01 CA220378, R01 CA221938, R01 CA158079-01, and U01 CA176110 funded co-author effort and data acquisition/analysis. Mayo Clinic Foundation provided funding for open access publication fees.

## Conflict of Interest

KSw was co-founder of Precision Oncology Insights, US Patent US8571844B2. KSc: financial interest—Imaging Biometrics LLC; ownership interest—IQ-AQ Ltd; ownership interest—Prism Clinical Imaging, Inc. LH: Imaging Biometrics (Medical Advisory Board); Precision Oncology Insights (co-founder), Bayer Pharmaceutical (paid speaker), US Patent Number 10,909,675.

The remaining authors declare that the research was conducted in the absence of any commercial or financial relationships that could be construed as a potential conflict of interest.

## Publisher's Note

All claims expressed in this article are solely those of the authors and do not necessarily represent those of their affiliated organizations, or those of the publisher, the editors and the reviewers. Any product that may be evaluated in this article, or claim that may be made by its manufacturer, is not guaranteed or endorsed by the publisher.

## References

[1] National Comprehensive Cancer Network. Central Nervous System Cancers. (Version 2.2021). Available online at: https://www.nccn.org/professionals/physician_gls/pdf/cns.pdf (accessed October 7, 2021).

[2] Ellingson BM Bendszus M Boxerman J Barboriak D Erickson BJ Smits M. Consensus recommendations for a standardized brain tumor imaging protocol in clinical trials. Neuro Oncol. (2015) 17:1188–98. 10.1093/neuonc/nov095 26250565 PMC4588759

[3] Verma N Cowperthwaite MC Burnett MG Markey MK. Differentiating tumor recurrence from treatment necrosis: a review of neuro-oncologic imaging strategies. Neuro Oncol. (2013) 15:515–34. 10.1093/neuonc/nos307 23325863 PMC3635510

[4] Walker MD Alexander E Jr Hunt WE MacCarty CS Mahaley MS Jr Mealey J Jr. Evaluation of BCNU and/or radiotherapy in the treatment of anaplastic gliomas. A cooperative clinical trial: a cooperative clinical trial. J Neurosurg. (1978) 49:333–43. 10.3171/jns.1978.49.3.0333 355604

[5] Stupp R Mason WP van den Bent MJ Weller M Fisher B Taphoorn MJB. Radiotherapy plus concomitant and adjuvant temozolomide for glioblastoma. N Engl J Med. (2005) 352:987–96. 10.1056/NEJMoa043330 15758009

[6] Fisher JP Adamson DC. Current FDA-approved therapies for high-grade malignant gliomas. Biomedicines. (2021) 9:324. 10.3390/biomedicines9030324 33810154 PMC8004675

[7] Wenger KJ Wagner M You S-J Franz K Harter PN Burger MC. Bevacizumab as a last-line treatment for glioblastoma following failure of radiotherapy, temozolomide and lomustine. Oncol Lett. (2017) 14:1141–6. 10.3892/ol.2017.6251 28693286 PMC5494648

[8] Brandsma D van den Bent MJ. Pseudoprogression and pseudoresponse in the treatment of gliomas. Curr Opin Neurol. (2009) 22:633–8. 10.1097/WCO.0b013e328332363e 19770760

[9] Xu S Tang L Li X Fan F Liu Z. Immunotherapy for glioma: current management and future application. Cancer Lett. (2020) 476:1–12. 10.1016/j.canlet.2020.02.002 32044356

[10] Huang B Li X Li Y Zhang J Zong Z Zhang H. Current immunotherapies for glioblastoma multiforme. Front Immunol. (2020) 11:603911. 10.3389/fimmu.2020.603911 33767690 PMC7986847

[11] Fecci PE Ochiai H Mitchell DA Grossi PM Sweeney AE Archer GE. Systemic CTLA-4 blockade ameliorates glioma-induced changes to the CD4+ T cell compartment without affecting regulatory T-cell function. Clin Cancer Res. (2007) 13:2158–67. 10.1158/1078-0432.CCR-06-2070 17404100

[12] Zeng J See AP Phallen J Jackson CM Belcaid Z Ruzevick J. Anti-PD-1 blockade and stereotactic radiation produce long-term survival in mice with intracranial gliomas. Int J Radiat Oncol Biol Phys. (2013) 86:343–9. 10.1016/j.ijrobp.2012.12.025 23462419 PMC3963403

[13] Wheeler CJ Black KL Liu G Mazer M Zhang X-X Pepkowitz S. Vaccination elicits correlated immune and clinical responses in glioblastoma multiforme patients. Cancer Res. (2008) 68:5955–64. 10.1158/0008-5472.CAN-07-5973 18632651

[14] Schaller TH Sampson JH. Advances and challenges: dendritic cell vaccination strategies for glioblastoma. Exp Rev Vaccines. (2017) 16:27–36. 10.1080/14760584.2016.1218762 27500911 PMC5479410

[15] Nehama D Di Ianni N Musio S Du H Patané M Pollo B. B7-H3-redirected chimeric antigen receptor T cells target glioblastoma and neurospheres. EBioMedicine. (2019) 47:33–43. 10.1016/j.ebiom.2019.08.030 31466914 PMC6796553

[16] Brown CE Alizadeh D Starr R Weng L Wagner JR Naranjo A. Regression of glioblastoma after chimeric antigen receptor T-cell therapy. N Engl J Med. (2016) 375:2561–9. 10.1056/NEJMoa1610497 28029927 PMC5390684

[17] Brandsma D Stalpers L Taal W Sminia P van den Bent MJ. Clinical features, mechanisms, and management of pseudoprogression in malignant gliomas. Lancet Oncol. (2008) 9:453–61. 10.1016/S1470-2045(08)70125-6 18452856

[18] Thust SC van den Bent MJ Smits M. Pseudoprogression of brain tumors: pseudoprogression of Brain Tumors. J Magn Reson Imaging. (2018) 48:571–89. 10.1002/jmri.26171 29734497 PMC6175399

[19] Ellingson BM Chung C Pope WB Boxerman JL Kaufmann TJ. Pseudoprogression, radionecrosis, inflammation or true tumor progression? Challenges associated with glioblastoma response assessment in an evolving therapeutic landscape. J Neurooncol. (2017) 134:495–504. 10.1007/s11060-017-2375-2 28382534 PMC7893814

[20] Miyatake S-I Nonoguchi N Furuse M Yoritsune E Miyata T Kawabata S. Pathophysiology, diagnosis, and treatment of radiation necrosis in the brain. Neurol Med Chir. (2015) 55:50–9. 10.2176/nmc.ra.2014-0188 25744350 PMC4533398

[21] de Wit MCY de Bruin HG Eijkenboom W Sillevis Smitt PAE van den Bent MJ. Immediate post-radiotherapy changes in malignant glioma can mimic tumor progression. Neurology. (2004) 63:535–7. 10.1212/01.wnl.0000133398.11870.9a 15304589

[22] Gerstner ER McNamara MB Norden AD Lafrankie D Wen PY. Effect of adding temozolomide to radiation therapy on the incidence of pseudo-progression. J Neurooncol. (2009) 94:97–101. 10.1007/s11060-009-9809-4 19221865

[23] Chamberlain MC Glantz MJ Chalmers L Van Horn A Sloan AE. Early necrosis following concurrent Temodar and radiotherapy in patients with glioblastoma. J Neurooncol. (2007) 82:81–3. 10.1007/s11060-006-9241-y 16944309

[24] Bobola MS Kolstoe DD Blank A Silber JR. Minimally cytotoxic doses of temozolomide produce radiosensitization in human glioblastoma cells regardless of MGMT expression. Mol Cancer Ther. (2010) 9:1208–18. 10.1158/1535-7163.MCT-10-0010 20457618 PMC2869471

[25] Gahramanov S Varallyay C Tyson RM Lacy C Fu R Netto JP. Diagnosis of pseudoprogression using MRI perfusion in patients with glioblastoma multiforme may predict improved survival. CNS Oncol. (2014) 3:389–400. 10.2217/cns.14.42 25438810 PMC4590772

[26] Bronk JK Guha-Thakurta N Allen PK Mahajan A Grosshans DR McGovern SL. Analysis of pseudoprogression after proton or photon therapy of 99 patients with low grade and anaplastic glioma. Clin Transl Radiat Oncol. (2018) 9:30–4. 10.1016/j.ctro.2018.01.002 29594248 PMC5862685

[27] Brandes AA Franceschi E Tosoni A Blatt V Pession A Tallini G. MGMT promoter methylation status can predict the incidence and outcome of pseudoprogression after concomitant radiochemotherapy in newly diagnosed glioblastoma patients. J Clin Oncol. (2008) 26:2192–7. 10.1200/JCO.2007.14.8163 18445844

[28] McGirt MJ Bulsara KR Cummings TJ New KC Little KM Friedman HS. Prognostic value of magnetic resonance imaging-guided stereotactic biopsy in the evaluation of recurrent malignant astrocytoma compared with a lesion due to radiation effect. J Neurosurg. (2003) 98:14–20. 10.3171/jns.2003.98.1.0014 12546347

[29] Himes BT Arnett AL Merrell KW Gates MJ Bhargav AG Raghunathan A. Glioblastoma recurrence versus treatment effect in a pathology-documented series. Can J Neurol Sci. (2020) 47:525–30. 10.1017/cjn.2020.36 32077389 PMC10807241

[30] Hygino da Cruz LC Jr Rodriguez I Domingues RC Gasparetto EL Sorensen AG. Pseudoprogression and pseudoresponse: imaging challenges in the assessment of posttreatment glioma. AJNR Am J Neuroradiol. (2011) 32:1978–85. 10.3174/ajnr.A2397 21393407 PMC7964401

[31] Melguizo-Gavilanes I Bruner JM Guha-Thakurta N Hess KR Puduvalli VK. Characterization of pseudoprogression in patients with glioblastoma: is histology the gold standard? J Neurooncol. (2015) 123:141–50. 10.1007/s11060-015-1774-5 25894594 PMC4780341

[32] Abbasi AW Westerlaan HE Holtman GA Aden KM van Laar PJ van der Hoorn A. Incidence of tumour progression and pseudoprogression in high-grade gliomas: a systematic review and meta-analysis. Clin Neuroradiol. (2018) 28:401–11. 10.1007/s00062-017-0584-x 28466127 PMC6105173

[33] Parvez K Parvez A Zadeh G. The diagnosis and treatment of pseudoprogression, radiation necrosis and brain tumor recurrence. Int J Mol Sci. (2014) 15:11832–46. 10.3390/ijms150711832 24995696 PMC4139817

[34] Song J Kadaba P Kravitz A Hormigo A Friedman J Belani P. Multiparametric MRI for early identification of therapeutic response in recurrent glioblastoma treated with immune checkpoint inhibitors. Neuro Oncol. (2020) 22:1658–66. 10.1093/neuonc/noaa066 32193547 PMC7846197

[35] Jia W Gao Q Han A Zhu H Yu J. The potential mechanism, recognition and clinical significance of tumor pseudoprogression after immunotherapy. Cancer Biol Med. (2019) 16:655–70. 10.20892/j.issn.2095-3941.2019.0144 31908886 PMC6936240

[36] Okada H Weller M Huang R Finocchiaro G Gilbert MR Wick W. Immunotherapy response assessment in neuro-oncology: a report of the RANO working group. Lancet Oncol. (2015) 16:e534–42. 10.1016/S1470-2045(15)00088-1 26545842 PMC4638131

[37] Cohen JV Alomari AK Vortmeyer AO Jilaveanu LB Goldberg SB Mahajan A. Melanoma brain metastasis pseudoprogression after pembrolizumab treatment. Cancer Immunol Res. (2016) 4:179–82. 10.1158/2326-6066.CIR-15-0160 26701266 PMC4881844

[38] Gatson NTN Makary M Bross SP Vadakara J Maiers T Mongelluzzo GJ. Case series review of neuroradiologic changes associated with immune checkpoint inhibitor therapy. Neurooncol Pract. (2021) 8:247–58. 10.1093/nop/npaa079 34055372 PMC8153815

[39] Johnson DB Manouchehri A Haugh AM Quach HT Balko JM Lebrun-Vignes B. Neurologic toxicity associated with immune checkpoint inhibitors: a pharmacovigilance study. J Immunother Cancer. (2019) 7:134. 10.1186/s40425-019-0617-x 31118078 PMC6530194

[40] Spain L Walls G Julve M O'Meara K Schmid T Kalaitzaki E. Neurotoxicity from immune-checkpoint inhibition in the treatment of melanoma: a single centre experience and review of the literature. Ann Oncol. (2017) 28:377–85. 10.1093/annonc/mdw558 28426103

[41] Albert FK Forsting M Sartor K Adams H-P Kunze S. Early postoperative magnetic resonance imaging after resection of malignant glioma: objective evaluation of residual tumor and its influence on regrowth and prognosis. Neurosurgery. (1994) 34:45–61. 10.1227/00006123-199401000-00008 8121569

[42] Macdonald DR Cascino TL Schold SC Jr Cairncross JG. Response criteria for phase II studies of supratentorial malignant glioma. J Clin Oncol. (1990) 8:1277–80. 10.1200/JCO.1990.8.7.1277 2358840

[43] Wen PY Macdonald DR Reardon DA Cloughesy TF Sorensen AG Galanis E. Updated response assessment criteria for high-grade gliomas: response assessment in neuro-oncology working group. J Clin Oncol. (2010) 28:1963–72. 10.1200/JCO.2009.26.3541 20231676

[44] Ellingson BM Wen PY Cloughesy TF. Modified criteria for radiographic response assessment in glioblastoma clinical trials. Neurotherapeutics. (2017) 14:307–20. 10.1007/s13311-016-0507-6 28108885 PMC5398984

[45] Dubois LG Campanati L Righy C D'Andrea-Meira I Spohr TCL de SE Porto-Carreiro I. Gliomas and the vascular fragility of the blood brain barrier. Front Cell Neurosci. (2014) 8:418. 10.3389/fncel.2014.00418 25565956 PMC4264502

[46] Hu LS Hawkins-Daarud A Wang L Li J Swanson KR. Imaging of intratumoral heterogeneity in high-grade glioma. Cancer Lett. (2020) 477:97–106. 10.1016/j.canlet.2020.02.025 32112907 PMC7108976

[47] Kumar AJ Leeds NE Fuller GN Van Tassel P Maor MH Sawaya RE. Malignant gliomas: MR imaging spectrum of radiation therapy- and chemotherapy-induced necrosis of the brain after treatment. Radiology. (2000) 217:377–84. 10.1148/radiology.217.2.r00nv36377 11058631

[48] Tihan T Barletta J Parney I Lamborn K Sneed PK Chang S. Prognostic value of detecting recurrent glioblastoma multiforme in surgical specimens from patients after radiotherapy: should pathology evaluation alter treatment decisions? Hum Pathol. (2006) 37:272–82. 10.1016/j.humpath.2005.11.010 16613322

[49] Hu LS Eschbacher JM Heiserman JE Dueck AC Shapiro WR Liu S. Reevaluating the imaging definition of tumor progression: perfusion MRI quantifies recurrent glioblastoma tumor fraction, pseudoprogression, and radiation necrosis to predict survival. Neuro Oncol. (2012) 14:919–30. 10.1093/neuonc/nos112 22561797 PMC3379799

[50] Winter SF Vaios EJ Muzikansky A Martinez-Lage M Bussière MR Shih HA. Defining treatment-related adverse effects in patients with glioma: distinctive features of pseudoprogression and treatment-induced necrosis. Oncologist. (2020) 25:e1221–32. 10.1634/theoncologist.2020-0085 32488924 PMC7418360

[51] Hoxworth JM Eschbacher JM Gonzales AC Singleton KW Leon GD Smith KA. Performance of standardized relative CBV for quantifying regional histologic tumor burden in recurrent high-grade glioma: comparison against normalized relative CBV using image-localized stereotactic biopsies. AJNR Am J Neuroradiol. (2020) 41:408–15. 10.3174/ajnr.A6486 32165359 PMC7077911

[52] Prah MA Al-Gizawiy MM Mueller WM Cochran EJ Hoffmann RG Connelly JM. Spatial discrimination of glioblastoma and treatment effect with histologically-validated perfusion and diffusion magnetic resonance imaging metrics. J Neurooncol. (2018) 136:13–21. 10.1007/s11060-017-2617-3 28900832 PMC5756123

[53] Furtado AD Ceschin R Blüml S Mason G Jakacki RI Okada H. Neuroimaging of peptide-based vaccine therapy in pediatric brain tumors: initial experience. Neuroimaging Clin N Am. (2017) 27:155–66. 10.1016/j.nic.2016.09.002 27889021 PMC5127439

[54] Forsyth PA Kelly PJ Cascino TL Scheithauer BW Shaw EG Dinapoli RP. Radiation necrosis or glioma recurrence: is computer-assisted stereotactic biopsy useful? J Neurosurg. (1995) 82:436–44. 10.3171/jns.1995.82.3.0436 7861222

[55] Bagley SJ Schwab RD Nelson E Viaene AN Binder ZA Lustig RA. Histopathologic quantification of viable tumor versus treatment effect in surgically resected recurrent glioblastoma. J Neurooncol. (2019) 141:421–9. 10.1007/s11060-018-03050-6 30446903

[56] Essig M Shiroishi MS Nguyen TB Saake M Provenzale JM Enterline D. Perfusion MRI: the five most frequently asked technical questions. AJR Am J Roentgenol. (2013) 200:24–34. 10.2214/AJR.12.9543 23255738 PMC3593114

[57] Boxerman JL Quarles CC Hu LS Erickson BJ Gerstner ER Smits M. Consensus recommendations for a dynamic susceptibility contrast MRI protocol for use in high-grade gliomas. Neuro Oncol. (2020) 22:1262–75. 10.1093/neuonc/noaa141 32516388 PMC7523451

[58] Cha S Lu S Johnson G Knopp EA. Dynamic susceptibility contrast MR imaging: correlation of signal intensity changes with cerebral blood volume measurements. J Magn Reson Imaging. (2000) 11:114–9. 10.1002/(sici)1522-2586(200002)11:2<114::aid-jmri6>3.0.co;2-s 10713942

[59] Cha S Lupo JM Chen M-H Lamborn KR McDermott MW Berger MS. Differentiation of glioblastoma multiforme and single brain metastasis by peak height and percentage of signal intensity recovery derived from dynamic susceptibility-weighted contrast-enhanced perfusion MR imaging. AJNR Am J Neuroradiol. (2007) 28:1078–84. 10.3174/ajnr.A0484 17569962 PMC8134129

[60] Hu LS Eschbacher JM Dueck AC Heiserman JE Liu S Karis JP. Correlations between perfusion MR imaging cerebral blood volume, microvessel quantification, and clinical outcome using stereotactic analysis in recurrent high-grade glioma. AJNR Am J Neuroradiol. (2012) 33:69–76. 10.3174/ajnr.A2743 22095961 PMC7966183

[61] Hu LS Baxter LC Smith KA Feuerstein BG Karis JP Eschbacher JM. Relative cerebral blood volume values to differentiate high-grade glioma recurrence from posttreatment radiation effect: direct correlation between image-guided tissue histopathology and localized dynamic susceptibility-weighted contrast-enhanced perfusion MR imaging measurements. AJNR Am J Neuroradiol. (2009) 30:552–8. 10.3174/ajnr.A1377 19056837 PMC7051449

[62] Mangla R Singh G Ziegelitz D Milano MT Korones DN Zhong J. Changes in relative cerebral blood volume 1 month after radiation-temozolomide therapy can help predict overall survival in patients with glioblastoma. Radiology. (2010) 256:575–84. 10.1148/radiol.10091440 20529987

[63] Alexiou GA Zikou A Tsiouris S Goussia A Kosta P Papadopoulos A. Comparison of diffusion tensor, dynamic susceptibility contrast MRI and (99m)Tc-Tetrofosmin brain SPECT for the detection of recurrent high-grade glioma. Magn Reson Imaging. (2014) 32:854–9. 10.1016/j.mri.2014.04.013 24848292

[64] Gasparetto EL Pawlak MA Patel SH Huse J Woo JH Krejza J. Posttreatment recurrence of malignant brain neoplasm: accuracy of relative cerebral blood volume fraction in discriminating low from high malignant histologic volume fraction. Radiology. (2009) 250:887–96. 10.1148/radiol.2502071444 19244052

[65] Martínez-Martínez A Martínez-Bosch J. Perfusion magnetic resonance imaging for high grade astrocytomas: can cerebral blood volume, peak height, and percentage of signal intensity recovery distinguish between progression and pseudoprogression? Radiol (Engl Ed). (2014) 56:35–43. 10.1016/j.rxeng.2014.02.003 23790618

[66] Young RJ Gupta A Shah AD Graber JJ Chan TA Zhang Z. MRI perfusion in determining pseudoprogression in patients with glioblastoma. Clin Imaging. (2013) 37:41–9. 10.1016/j.clinimag.2012.02.016 23151413 PMC4755513

[67] Kim HS Suh CH Kim N Choi C-G Kim SJ. Histogram analysis of intravoxel incoherent motion for differentiating recurrent tumor from treatment effect in patients with glioblastoma: initial clinical experience. AJNR Am J Neuroradiol. (2014) 35:490–7. 10.3174/ajnr.A3719 23969343 PMC7964718

[68] Patel P Baradaran H Delgado D Askin G Christos P John Tsiouris A. MR perfusion-weighted imaging in the evaluation of high-grade gliomas after treatment: a systematic review and meta-analysis. Neuro Oncol. (2017) 19:118–27. 10.1093/neuonc/now148 27502247 PMC5193025

[69] Seeger A Braun C Skardelly M Paulsen F Schittenhelm J Ernemann U. Comparison of three different MR perfusion techniques and MR spectroscopy for multiparametric assessment in distinguishing recurrent high-grade gliomas from stable disease. Acad Radiol. (2013) 20:1557–65. 10.1016/j.acra.2013.09.003 24200483

[70] Sugahara T Korogi Y Tomiguchi S Shigematsu Y Ikushima I Kira T. Posttherapeutic intraaxial brain tumor: the value of perfusion-sensitive contrast-enhanced MR imaging for differentiating tumor recurrence from nonneoplastic contrast-enhancing tissue. AJNR Am J Neuroradiol. (2000) 21:901–9. 10815666 PMC7976740

[71] Prager AJ Martinez N Beal K Omuro A Zhang Z Young RJ. Diffusion and perfusion MRI to differentiate treatment-related changes including pseudoprogression from recurrent tumors in high-grade gliomas with histopathologic evidence. AJNR Am J Neuroradiol. (2015) 36:877–85. 10.3174/ajnr.A4218 25593202 PMC4731220

[72] Barajas RF Jr Chang JS Segal MR Parsa AT McDermott MW Berger MS. Differentiation of recurrent glioblastoma multiforme from radiation necrosis after external beam radiation therapy with dynamic susceptibility-weighted contrast-enhanced perfusion MR imaging. Radiology. (2009) 253:486–96. 10.1148/radiol.2532090007 19789240 PMC2770116

[73] Iv M Liu X Lavezo J Gentles AJ Ghanem R Lummus S. Perfusion MRI-based fractional tumor burden differentiates between tumor and treatment effect in recurrent glioblastomas and informs clinical decision-making. AJNR Am J Neuroradiol. (2019) 40:1649–57. 10.3174/ajnr.A6211 31515215 PMC7028562

[74] Boxerman JL Schmainda KM Weisskoff RM. Relative cerebral blood volume maps corrected for contrast agent extravasation significantly correlate with glioma tumor grade, whereas uncorrected maps do not. AJNR Am J Neuroradiol. (2006) 27:859–67. 16611779 PMC8134002

[75] Boxerman JL Prah DE Paulson ES Machan JT Bedekar D Schmainda KM. The Role of preload and leakage correction in gadolinium-based cerebral blood volume estimation determined by comparison with MION as a criterion standard. AJNR Am J Neuroradiol. (2012) 33:1081–7. 10.3174/ajnr.A2934 22322605 PMC4331024

[76] Schmainda KM Rand SD Joseph AM Lund R Ward BD Pathak AP. Characterization of a first-pass gradient-echo spin-echo method to predict brain tumor grade and angiogenesis. AJNR Am J Neuroradiol. (2004) 25:1524–32. 15502131 PMC7976425

[77] Hu LS Baxter LC Pinnaduwage DS Paine TL Karis JP Feuerstein BG. Optimized preload leakage-correction methods to improve the diagnostic accuracy of dynamic susceptibility-weighted contrast-enhanced perfusion MR imaging in posttreatment gliomas. AJNR Am J Neuroradiol. (2010) 31:40–8. 10.3174/ajnr.A1787 19749223 PMC4323177

[78] Welker K Boxerman J Kalnin A Kaufmann T Shiroishi M Wintermark M. ASFNR recommendations for clinical performance of MR dynamic susceptibility contrast perfusion imaging of the brain. AJNR Am J Neuroradiol. (2015) 36:E41–51. 10.3174/ajnr.A4341 25907520 PMC5074767

[79] Semmineh NB Bell LC Stokes AM Hu LS Boxerman JL Quarles CC. Optimization of acquisition and analysis methods for clinical dynamic susceptibility contrast MRI using a population-based digital reference object. AJNR Am J Neuroradiol. (2018) 39:1981–8. 10.3174/ajnr.A5827 30309842 PMC6239921

[80] Conte GM Castellano A Altabella L Iadanza A Cadioli M Falini A. Reproducibility of dynamic contrast-enhanced MRI and dynamic susceptibility contrast MRI in the study of brain gliomas: a comparison of data obtained using different commercial software. Radiol Med. (2017) 122:294–302. 10.1007/s11547-016-0720-8 28070841

[81] Hu LS Kelm Z Korfiatis P Dueck AC Elrod C Ellingson BM. Impact of software modeling on the accuracy of perfusion MRI in glioma. AJNR Am J Neuroradiol. (2015) 36:2242–9. 10.3174/ajnr.A4451 26359151 PMC4681640

[82] Bell LC Semmineh N An H Eldeniz C Wahl R Schmainda KM. Evaluating the use of rCBV as a tumor grade and treatment response classifier across NCI quantitative imaging network sites: part II of the DSC-MRI digital reference object (DRO) challenge. Tomography. (2020) 6:203–8. 10.18383/j.tom.2020.00012 32548297 PMC7289259

[83] Schmainda KM Prah MA Rand SD Liu Y Logan B Muzi M. Multisite concordance of DSC-MRI analysis for brain tumors: results of a national cancer institute quantitative imaging network collaborative project. AJNR Am J Neuroradiol. (2018) 39:1008–16. 10.3174/ajnr.A5675 29794239 PMC6002911

[84] Bell LC Semmineh N An H Eldeniz C Wahl R Schmainda KM. Evaluating multisite rCBV consistency from DSC-MRI imaging protocols and postprocessing software across the NCI Quantitative Imaging Network sites using a digital reference object (DRO). Tomography. (2019) 5:110–7. 10.18383/j.tom.2018.00041 30854448 PMC6403027

[85] Bedekar D Jensen T Schmainda KM. Standardization of relative cerebral blood volume (rCBV) image maps for ease of both inter- and intrapatient comparisons. Magn Reson Med. (2010) 64:907–13. 10.1002/mrm.22445 20806381 PMC4323176

[86] Winkler F Kozin SV Tong RT Chae S-S Booth MF Garkavtsev I. Kinetics of vascular normalization by VEGFR2 blockade governs brain tumor response to radiation. Cancer Cell. (2004) 6:553–63. 10.1016/j.ccr.2004.10.011 15607960

[87] Batchelor TT Sorensen AG di Tomaso E Zhang W-T Duda DG Cohen KS. AZD2171, a pan-VEGF receptor tyrosine kinase inhibitor, normalizes tumor vasculature and alleviates edema in glioblastoma patients. Cancer Cell. (2007) 11:83–95. 10.1016/j.ccr.2006.11.021 17222792 PMC2748664

[88] Schmainda KM Prah M Connelly J Rand SD Hoffman RG Mueller W. Dynamic-susceptibility contrast agent MRI measures of relative cerebral blood volume predict response to bevacizumab in recurrent high-grade glioma. Neuro Oncol. (2014) 16:880–8. 10.1093/neuonc/not216 24431219 PMC4022214

[89] Cuccarini V Aquino D Gioppo A Anghileri E Pellegatta S Schettino C. Advanced MRI assessment during dendritic cell immunotherapy added to standard treatment against glioblastoma. J Clin Med. (2019) 8:2007. 10.3390/jcm8112007 31744235 PMC6912338

[90] Qin L Li X Stroiney A Qu J Helgager J Reardon DA. Advanced MRI assessment to predict benefit of anti-programmed cell death 1 protein immunotherapy response in patients with recurrent glioblastoma. Neuroradiology. (2017) 59:135–45. 10.1007/s00234-016-1769-8 28070598 PMC6097616

[91] Neuwelt EA Várallyay CG Manninger S Solymosi D Haluska M Hunt MA. The potential of ferumoxytol nanoparticle magnetic resonance imaging, perfusion, and angiography in central nervous system malignancy: a pilot study. Neurosurgery. (2007) 60:601–11; discussion 611–2. 10.1227/01.NEU.0000255350.71700.37 17415196

[92] Gahramanov S Raslan AM Muldoon LL Hamilton BE Rooney WD Varallyay CG. Potential for differentiation of pseudoprogression from true tumor progression with dynamic susceptibility-weighted contrast-enhanced magnetic resonance imaging using ferumoxytol vs. gadoteridol: a pilot study Int J Radiat Oncol Biol Phys. (2011) 79:514–23. 10.1016/j.ijrobp.2009.10.072 20395065 PMC3111452

[93] Toth GB Varallyay CG Horvath A Bashir MR Choyke PL Daldrup-Link HE. Current and potential imaging applications of ferumoxytol for magnetic resonance imaging. Kidney Int. (2017) 92:47–66. 10.1016/j.kint.2016.12.037 28434822 PMC5505659

[94] Varallyay CG Muldoon LL Gahramanov S Wu YJ Goodman JA Li X. Dynamic MRI using iron oxide nanoparticles to assess early vascular effects of antiangiogenic versus corticosteroid treatment in a glioma model. J Cereb Blood Flow Metab. (2009) 29:853–60. 10.1038/jcbfm.2008.162 19142191 PMC2747492

[95] McConnell HL Schwartz DL Richardson BE Woltjer RL Muldoon LL Neuwelt EA. Ferumoxytol nanoparticle uptake in brain during acute neuroinflammation is cell-specific. Nanomedicine. (2016) 12:1535–42. 10.1016/j.nano.2016.03.009 27071335 PMC4955720

[96] Barajas RF Hamilton BE Schwartz D McConnell HL Pettersson DR Horvath A. Combined iron oxide nanoparticle ferumoxytol and gadolinium contrast enhanced MRI define glioblastoma pseudoprogression. Neuro Oncol. (2019) 21:517–26. 10.1093/neuonc/noy160 30277536 PMC6422439

[97] Vasanawala SS Nguyen K-L Hope MD Bridges MD Hope TA Reeder SB. Safety and technique of ferumoxytol administration for MRI: safety and Technique of Ferumoxytol Administration for MRI. Magn Reson Med. (2016) 75:2107–11. 10.1002/mrm.26151 26890830 PMC4854636

[98] Bell LC Hu LS Stokes AM McGee SC Baxter LC Quarles CC. Characterizing the influence of preload dosing on percent signal recovery (PSR) and cerebral blood volume (CBV) measurements in a patient population with high-grade glioma using dynamic susceptibility contrast MRI. Tomography. (2017) 3:89–95. 10.18383/j.tom.2017.00004 28825039 PMC5557059

[99] Morabito R Alafaci C Pergolizzi S Pontoriero A Iati' G Bonanno L. DCE and DSC perfusion MRI diagnostic accuracy in the follow-up of primary and metastatic intra-axial brain tumors treated by radiosurgery with cyberknife. Radiat Oncol. (2019) 14:65. 10.1186/s13014-019-1271-7 30992043 PMC6466652

[100] Hamilton JD Lin J Ison C Leeds NE Jackson EF Fuller GN. Dynamic contrast-enhanced perfusion processing for neuroradiologists: model-dependent analysis may not be necessary for determining recurrent high-grade glioma versus treatment effect. AJNR Am J Neuroradiol. (2015) 36:686–93. 10.3174/ajnr.A4190 25500312 PMC7964311

[101] Bisdas S Naegele T Ritz R Dimostheni A Pfannenberg C Reimold M. Distinguishing recurrent high-grade gliomas from radiation injury: a pilot study using dynamic contrast-enhanced MR imaging. Acad Radiol. (2011) 18:575–83. 10.1016/j.acra.2011.01.018 21419671

[102] Barboriak DP Zhang Z Desai P Snyder BS Safriel Y McKinstry RC. Interreader variability of dynamic contrast-enhanced MRI of recurrent glioblastoma: the multicenter ACRIN 6677/RTOG 0625 study. Radiology. (2019) 290:467–76. 10.1148/radiol.2019181296 30480488 PMC6358054

[103] Anzalone N Castellano A Cadioli M Conte GM Cuccarini V Bizzi A. Brain gliomas: multicenter standardized assessment of dynamic contrast-enhanced and dynamic susceptibility contrast MR images. Radiology. (2018) 287:933–43. 10.1148/radiol.2017170362 29361245

[104] Petcharunpaisan S Ramalho J Castillo M. Arterial spin labeling in neuroimaging. World J Radiol. (2010) 2:384–98. 10.4329/wjr.v2.i10.384 21161024 PMC2999014

[105] Jovanovic M Radenkovic S Stosic-Opincal T Lavrnic S Gavrilovic S Lazovic-Popovic B. Differentiation between progression and pseudoprogresion by arterial spin labeling MRI in patients with glioblastoma multiforme. J BUON. (2017) 22:1061–7. 28952228

[106] Nyberg E Honce J Kleinschmidt-DeMasters BK Shukri B Kreidler S Nagae L. Arterial spin labeling: pathologically proven superiority over conventional MRI for detection of high-grade glioma progression after treatment. Neuroradiol J. (2016) 29:377–83. 10.1177/1971400916665375 27542895 PMC5033098

[107] Ozsunar Y Mullins ME Kwong K Hochberg FH Ament C Schaefer PW. Glioma recurrence versus radiation necrosis? A pilot comparison of arterial spin-labeled, dynamic susceptibility contrast enhanced MRI, and FDG-PET imaging. Acad Radiol. (2010) 17:282–90. 10.1016/j.acra.2009.10.024 20060750

[108] Choi YJ Kim HS Jahng G-H Kim SJ Suh DC. Pseudoprogression in patients with glioblastoma: added value of arterial spin labeling to dynamic susceptibility contrast perfusion MR imaging. Acta Radiol. (2013) 54:448–54. 10.1177/0284185112474916 23592805

[109] Ippolito L Morandi A Giannoni E Chiarugi P. Lactate: a metabolic driver in the tumour landscape. Trends Biochem Sci. (2019) 44:153–66. 10.1016/j.tibs.2018.10.011 30473428

[110] Choi C Ganji SK DeBerardinis RJ Hatanpaa KJ Rakheja D Kovacs Z. 2-hydroxyglutarate detection by magnetic resonance spectroscopy in IDH-mutated patients with gliomas. Nat Med. (2012) 18:624–9. 10.1038/nm.2682 22281806 PMC3615719

[111] Croteau D Scarpace L Hearshen D Gutierrez J Fisher JL Rock JP. Correlation between magnetic resonance spectroscopy imaging and image-guided biopsies: semiquantitative and qualitative histopathological analyses of patients with untreated glioma. Neurosurgery. (2001) 49:823–9. 10.1227/00006123-200110000-00008 11564242

[112] Rock JP Hearshen D Scarpace L Croteau D Gutierrez J Fisher JL. Correlations between magnetic resonance spectroscopy and image-guided histopathology, with special attention to radiation necrosis. Neurosurgery. (2002) 51:912–9; discussion 919-20. 10.1097/00006123-200210000-00010 12234397

[113] Rock JP Scarpace L Hearshen D Gutierrez J Fisher JL Rosenblum M. Associations among magnetic resonance spectroscopy, apparent diffusion coefficients, and image-guided histopathology with special attention to radiation necrosis. Neurosurgery. (2004) 54:1111–7; discussion 1117–9. 10.1227/01.neu.0000119328.56431.a7 15113465

[114] Fink JR Carr RB Matsusue E Iyer RS Rockhill JK Haynor DR. Comparison of 3 Tesla proton MR spectroscopy, MR perfusion and MR diffusion for distinguishing glioma recurrence from posttreatment effects. J Magn Reson Imaging. (2012) 35:56–63. 10.1002/jmri.22801 22002882

[115] Hu LS Wang L Hawkins-Daarud A Eschbacher JM Singleton KW Jackson PR. Uncertainty quantification in the radiogenomics modeling of EGFR amplification in glioblastoma. Sci Rep. (2021) 11:3932. 10.1038/s41598-021-83141-z 33594116 PMC7886858

[116] Hu LS Ning S Eschbacher JM Baxter LC Gaw N Ranjbar S. Radiogenomics to characterize regional genetic heterogeneity in glioblastoma. Neuro Oncol. (2017) 19:128–37. 10.1093/neuonc/now135 27502248 PMC5193022

[117] Hu LS Ning S Eschbacher JM Gaw N Dueck AC Smith KA. Multi-parametric MRI and texture analysis to visualize spatial histologic heterogeneity and tumor extent in Glioblastoma. PLoS One. (2015) 10:e0141506. 10.1371/journal.pone.0141506 26599106 PMC4658019

[118] Akbari H Rathore S Bakas S Nasrallah MP Shukla G Mamourian E. Histopathology-validated machine learning radiographic biomarker for noninvasive discrimination between true progression and pseudo-progression in glioblastoma. Cancer. (2020) 126:2625–36. 10.1002/cncr.32790 32129893 PMC7893811

[119] Gao Y Xiao X Han B Li G Ning X Wang D. Deep learning methodology for differentiating glioma recurrence from radiation necrosis using multimodal magnetic resonance imaging: algorithm development and validation (preprint). JMIR Preprints. (2020). 10.2196/preprints.19805 PMC7708085 33200991

[120] Zach L Guez D Last D Daniels D Grober Y Nissim O. Delayed contrast extravasation MRI: a new paradigm in neuro-oncology. Neuro Oncol. (2015) 17:457–65. 10.1093/neuonc/nou230 25452395 PMC4483101

